# OVERVIEW OF THE EMF 32 STUDY ON U.S. CARBON TAX SCENARIOS*

**DOI:** 10.1142/S201000781840002X

**Published:** 2018-03-20

**Authors:** JAMES R. MCFARLAND, ALLEN A. FAWCETT, ADELE C. MORRIS, JOHN M. REILLY, PETER J. WILCOXEN

**Affiliations:** † U.S. Environmental Protection Agency 1200 Pennsylvania Avenue NW Washington, DC 20460, USA; ‡ Brookings Institution, 1775 Massachusetts Ave NW Washington, DC 20036, USA; § Joint Program on the Science and Policy of Global Change Massachusetts Institute of Technology 77 Massachusetts Avenue, Cambridge, MA 02139, USA; ¶ Maxwell School Syracuse University and Brookings, NY, USA

**Keywords:** Carbon tax, revenue recycling, model comparison, climate change, CGE models

## Abstract

The Energy Modeling Forum (EMF) 32 study on carbon tax scenarios analyzed a set of illustrative policies in the United States that place an economy-wide tax on fossil-fuel-related carbon dioxide (CO_2_) emissions, a carbon tax for short. Eleven modeling teams ran these stylized scenarios, which vary by the initial carbon tax rate, the rate at which the tax escalates over time, and the use of the revenues. Modelers reported their results for the effects of the policies, relative to a reference scenario that does not include a carbon tax, on emissions, economic activity, and outcomes within the U.S. energy system. This paper explains the scenario design, presents an overview of the results, and compares results from the participating models. In particular, we compare various outcomes across the models, such as emissions, revenue, gross domestic product, sectoral impacts, and welfare.

## 1. Introduction

The Stanford Energy Modeling Forum (EMF) was established in 1977 as an organization to convene international working groups of modelers and model users in energy, economics, and the environment to undertake specific studies, identify important issues, and share insights. The goal of the EMF is “modeling for insights, not for numbers.” In other words, identify policy-relevant insights and analyses that are robust across a wide range of models, provide explanations for differences in results from different models where possible, and identify high priority areas for future research. The EMF 32 study on carbon tax scenarios continues a long tradition of EMF studies that have explored climate policy issues.^1^

This study gathered together 11 modeling teams to run a common set of carbon tax scenarios, in order to explore how environmental and economic outcomes depend on the initial carbon tax rates, the rate at which the tax escalates, and how the government uses the revenue collected by the carbon tax. The study is also motivated by a desire to inform critical policy design decisions with information such as: the policies’ expected revenue and tax rate outcomes; options for balancing distributional and efficiency goals; the likely significance of international emissions leakage and competitiveness effects; and the outcomes of an emissions tax approach relative to regulation under the Clean Air Act. A key objective of this model inter-comparison project is to understand which insights are robust across models and scenarios and which are more sensitive.

This study provides timely analysis for public policy debates. Despite the skepticism from critics, recent polls suggest as many as two-thirds of those who voted for President Trump support regulating or taxing greenhouse gas (GHG) emissions.^2,3^ A number of groups and organizations, including some that are Republican, Libertarian, or conservative, have promoted the idea of a revenue-neutral carbon tax, with a variety of perspectives on how revenue neutrality should work in practice. Of course, a primary reason for implementing a carbon or GHG tax is to reduce emissions, but the revenue can serve other goals. The revenue could reduce the federal deficit, help finance tax reform, support new spending on infrastructure or other priorities, or provide rebates to households. For example, a group of senior Republican leaders have proposed a $40 per ton CO_2_ tax rising over time, with revenues rebated to U.S. families through a monthly dividend.^4^

As policymakers contemplate a carbon tax, potentially in the broader context of other potential fiscal reforms, this EMF study seeks to inform questions such as: How much revenue would different carbon tax trajectories generate? How will revenues change over time? How do economic outcomes depend on how the revenues are used? What might be the effect of a carbon tax on revenue from other taxes? There are 15 papers in this EMF 32 special issue. The introduction in Fawcett *et al.* (this issue) provides background on the history of the EMF and enumerates the papers in this collection. This paper provides a technical overview of the core modeling results, and Barron *et al.* (this issue) summarize the policy-relevant insights. Caron *et al.* (this issue) report details of the distributional outcomes of the policies, and Macaluso *et al.* (this issue) detail outcomes in the U.S. energy sector and other industries. The other 10 papers are written by individual modeling teams in the project, highlighting outcomes of interest in their work.

This paper proceeds as follows. Section 2 below reviews the overall study design, including the reference and policy scenarios and relevant modeling assumptions. Section 3 discusses the results of the four core policy scenarios. It reviews the scenarios’ emissions reductions, revenue raised, macroeconomic, sectoral, and welfare outcomes. Section 4 discusses how and why model results differ. Section 5 concludes with directions for future research. The supplementary material includes supplementary figures and additional technical discussion.

## 2. Overview of the Study Design

### 2.1. Scenario design

The EMF 32 modeling project consists of one reference scenario and several policy scenarios. The reference (or baseline) scenario projects a future for emissions and economic activity without new climate policy or GHG regulations on stationary sources by the U.S. Environmental Protection Agency (EPA). The policy scenarios impose different designs of a carbon tax in the United States. All of the scenarios are coordinated across models to the extent feasible. That means that the teams harmonized their baseline economic projections and policy representations so that the differences in their results primarily derive from the difference in models rather than differences in economic forecasts and policy implementation.

#### 2.1.1. The reference scenario

To the extent feasible, modelers included in their reference scenario any existing energy or related policies that might influence GHG emissions, such as the production and investment tax credits passed in December 2015. Also to the extent feasible, modelers calibrated their baseline to the Energy Information Administration’s Annual Energy Outlook (EIA AEO) Early Release, No Clean Power Plan case from April 2016 (U.S. EIA, 2016). The global models in the study also project a no-new-policy baseline for other countries and regions.

In both the reference and policy scenarios, we assume that policymakers will start to rein in annual deficits when the federal debt reaches 100% of GDP. Accordingly, the scenarios impose lump-sum taxes annually such that the debt to GDP ratio is no higher than one.

To be sure, the recent tax legislation in the United States changes the outlook relative to the 2016 AEO, including a number of changes to both the tax base and tax rates of corporate and personal income taxes. Deficits are now projected to amount to over $1 trillion higher over the next 10 years, and corporate statutory rates are significantly lower. We discuss the implications of these changes in Sec. 5 in the context of directions of future research.

#### 2.1.2. Carbon tax policy scenarios

The carbon tax scenarios vary over two key dimensions: the tax rate trajectory and the disposition of the revenue. We develop a tractable set of illustrative scenarios, as shown in the matrix in Table 1 below. The rows of the scenario matrix represent the carbon tax trajectories, and the columns represent how the revenue from the carbon tax is used or recycled. Each cell of the scenario matrix corresponds to a scenario in this study.

The orange cell at the top is the reference scenario, which has no carbon tax and thus has no carbon tax revenue to use. The 12 blue cells immediately below the reference scenario represent the “core” scenarios, simple illustrative carbon tax trajectories starting in 2020 at $25 or $50 (in 2010 dollars), rising at 1% or 5% annually, with the carbon tax revenue used to fund lump-sum rebates to households (*HH*), a reduction of the capital income tax rate (*K*), or a reduction of the labor income tax rate (*L*). These are the scenarios required of all the models participating in this study and are the primary focus of this overview paper.

The green cells to the right of the core scenarios are scenarios with alternative uses of the carbon tax revenue that: (1) devote half the net revenue to a capital income tax reduction and half to lump-sum rebates for households (½ *K* and ½ *HH*) or (2) achieve a certain constrained optimization goal for distributional outcomes through a combination of lump-sum rebates to low income households and reductions in the capital and labor tax rates (Low Inc. *HH*, *K*, *L*). For completeness, we list these scenarios here, but discuss the results in Caron *et al.* in this Special Issue, which focusses on the distributional outcomes of the EMF 32 exercise.

Tables 2 and 3 summarize the core policy scenarios.

The yellow cells at the bottom of the matrix are scenarios in which each model solves for the carbon tax trajectory that meets a particular environmental target. One solves for the carbon tax imposed solely on fossil carbon used in electricity production that achieves the electricity sector emissions level in 2030 that EPA projected under the agency’s Clean Power Plan as finalized in 2015. Another scenario solves for the carbon tax path that achieves a 26% reduction in economy-wide fossil CO_2_ emissions in 2025 relative to 2005 levels, a target consistent with the U.S. Nationally Determined Contribution submitted under the Paris Accord (UNFCCC, 2016), and a related scenario extends that to also achieve an 80% reduction by 2050, consistent with the G8 Major Economies Forum commitments in 2009. We call these the “solve-to-match” scenarios.

**Figure 1.**
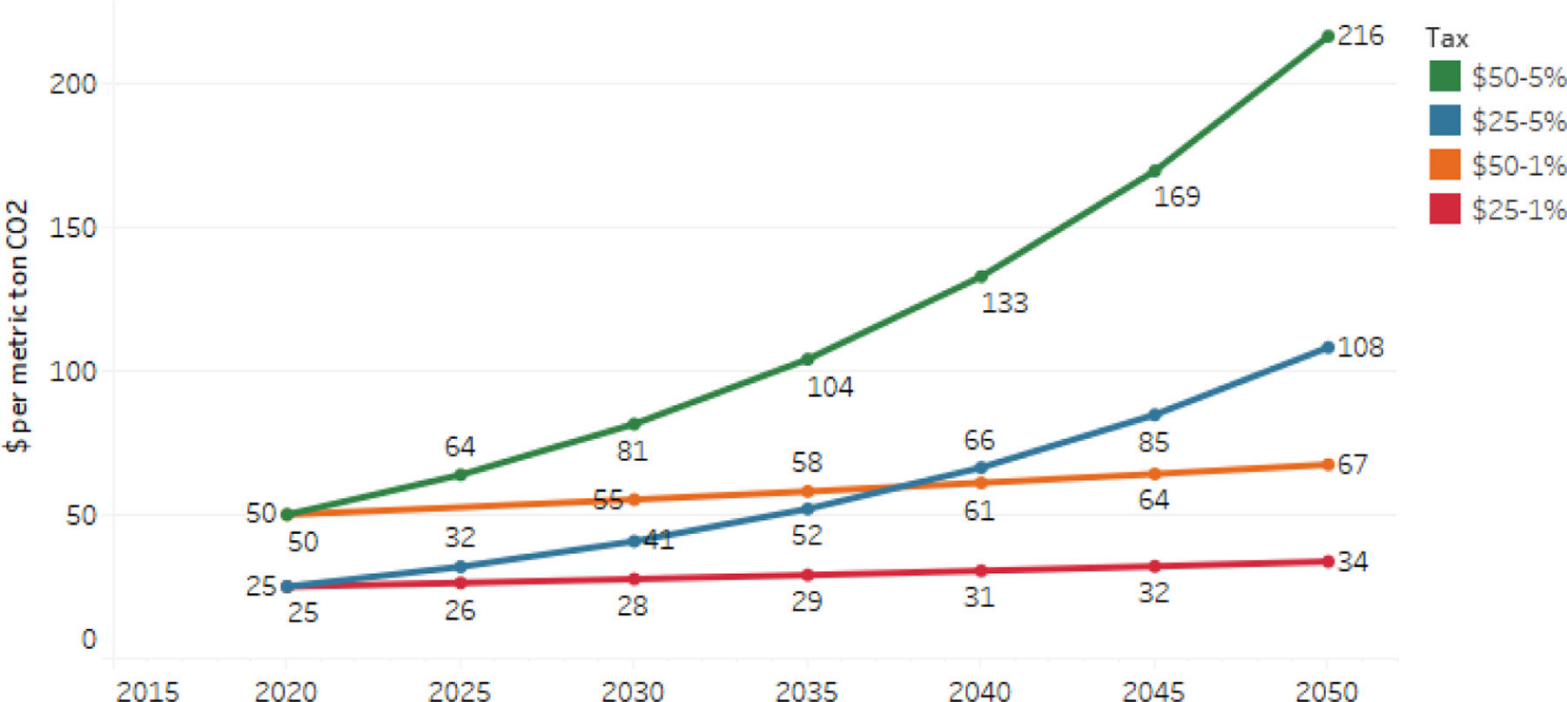
Tax rates from 2015 to 2050 in core scenarios ($2010 metric ton of CO_2_).

Each policy scenario imposes a carbon tax that begins in 2020 and increases annually until 2050. In years after 2050, we hold the carbon tax rate constant at its 2050 level. To facilitate comparison across models, we apply the tax only to fossil energy CO_2_.^5^ For the core scenarios, we model two initial tax rates ($25 and $50) and two rates of annual increase in the tax over inflation (1% and 5%), for a total of four different core tax rate trajectories, as shown in Fig. 1.

In all of the scenarios, we assume that international policy follows baseline levels of no new climate policy. These scenarios also assume no international or domestic offsets (credits for reductions outside of the taxed sectors), nor do they include border carbon adjustments, meaning that there is no charge on carbon-intensive goods (other than primary fossil fuels) imported into the United States or rebate for carbon taxes for carbon-intensive goods (again, other than primary fuels) exported from the United States.^6^ To the extent models allow, the scenarios apply a tax credit at the going rate to carbon sequestered with carbon capture and storage (CCS) technology. Most modelers assumed that the carbon tax is anticipated, not a surprise.

#### 2.1.3. Revenue recycling assumptions

As earlier research has shown, assumptions about how government spending changes (or not) because of a carbon tax have important implications for consumption-based measures of household welfare. That is because a carbon tax policy can change wages. If we hold the quantity of labor demanded by the government constant (a common assumption) and wages fall, then the carbon tax induces lower government spending on labor, and this in turn lowers total government consumption and the burden of government. In theory, some of the revenue recycling approaches could amplify this wage effect, such as via reductions in labor income taxes. This effect of the carbon tax on household welfare does not arise directly from the carbon tax but rather from its indirect effects on the overall size of government. Accordingly, in these scenarios, we hold government total real spending on *everything* (including interest payments) to baseline levels in all of the deficit-neutral scenarios.

In the household rebate scenarios, we recycle the revenue to households in lump-sum fashion, meaning equal rebates to all. We calculate the total rebate so that the federal deficit is unchanged relative to baseline. In the tax swap scenarios, we calculate the change in the capital or labor income tax rate (in percentage points) achievable in each period, using the recycled revenue to hold the deficit constant relative to baseline. Different models have different levels of aggregation of tax instruments, so readers should turn to the individual modeling papers (this issue) for discussion of how teams modeled their tax reductions. For example, the G-Cubed model has a single amalgamated labor income tax, which incorporates both payroll taxes and personal income tax on earned income, and a single amalgamated capital income tax, which incorporates taxes on corporate income and personal income that derives from capital gains, dividends, and interest. In contrast, the E3 model separately models separate revenue streams such as dividend income, capital gains, and interest income.

Models that allow for different tax brackets across household income classes apply an equal percentage point reduction in all brackets.

### 2.2. Models

Eleven modeling groups participated in the carbon tax study. Although 10 of the models are computable general equilibrium economic models, the models all differ in ways that have implications for results (Table 4). Models vary in the regional representation of the United States, ranging from a single region up to nine regions. Some of the models have global coverage, with a set of regions representing other countries. A few of the models have spatial detail in the electric power sector. Models vary in their sectoral coverage and range from roughly 10 sectors up to nearly 40, and they vary in whether they include only fossil-related CO_2_ or a broader set of GHGs. A few models report emissions of non-GHG air pollutants such as sulfur dioxide (SO_2_), nitrogen oxides (NO_*x*_), and mercury (Hg). Some models include representation of CCS technologies, and some have biomass fuels for electricity generation. Papers by individual modeling teams (this issue) provide some more model detail.

### 2.3. Limitations

A number of important qualifications apply to this study, as discussed in more detail in Barron *et al.* (this issue). Perhaps most importantly, the results do not quantify the potential economic and welfare benefits of lower CO_2_ emissions. Indeed, the primary goal of a price on carbon is to lower emissions that can disrupt the climate, acidify the ocean, and damage human health, economic activity, and the environment. A complete measure of welfare would account for the benefits of reducing those damages, along with the co-benefits from reduced conventional air pollution, such as SO_2_ and particulate matter. For an extensive discussion of many of the ways that climate change can impact the United States, see EPA (2015), “Climate Change in the United States: Benefits of Global Action.”

The EMF 32 study and the participating models also involve many of the same simplifying assumptions and limitations that apply to any study of this kind. For example, we assume no uncertainty about the persistence of the policies, when in reality, policymakers can abruptly rescind or tighten policies. We assume that the rest of the domestic and global policy landscapes are unchanged, and we ignore the potential for state policies to interact with federal policies. Some of the models include economic agents with an unrealistic degree of foresight, and we abstract from other inefficiencies (financing constraints, monopolistic competition, undersupplied research and development, and behavioral barriers) that could affect the outcome of a carbon tax in practice. In addition, the models do not represent rapidly developing technologies like electric vehicles that may play important roles in decarbonizing the economy over the long run.

The CGE models included in this exercise are “full employment” models, which assume that wages adjust (in some models gradually) such that total labor supply equals total labor demand. That means the study does not fully account for potential dislocations of individual workers and unemployment in certain sectors. Though understanding these labor market effects is important for the short run, our analysis emphasizes long-term changes to the macroeconomy. Some models may leave out new technologies or shifts in behavior that have already occurred owing to lags in the data used for their calibration, and none of the models includes an explicit representation of the research, development, and deployment process that will be critical to achieving deep decarbonization targets. In addition, some of the results are hard to compare across models because of underlying differences in the models’ structures. For example, models represent different tax instruments differently, and some include state-level taxes while others do not. We note in the text where this issue arises.

Because not all of the models include GHG emissions from nonfossil sources, this study only investigates controlling CO_2_ that derives from fossil fuel combustion. This means that we are not investigating potential abatement of important sources of GHGs such as methane from agriculture, landfill, mining, and upstream oil and gas sources. We also leave out carbon emissions from terrestrial sources like forests and agricultural soils and industrial processes like cement manufacturing. All of these GHG sources and sinks are important to the overall challenge of emissions reductions and can have significantly different costs and benefits, policy implementation options, and distributional considerations than controlling fossil CO_2_ emissions.

Finally, our scenarios assume unilateral action by the United States. Thus, we abstract from a significant goal of U.S. action: the potential to engage with other countries in the pursuit of multilateral climate change mitigation. Careful diplomacy and technological spillovers could greatly amplify the climate benefits of a price on carbon in the United States.

Despite these limitations, this work makes at least two important contributions. First, although our models’ projections may turn out to be off the mark in retrospect, *exante* they give decision makers far better insights than alternative tools to analyze options. Secondly, most modeling studies in the literature are individual modeling exercises using study-specific reference scenarios and policy assumptions. This makes it hard to assess which results in the literature are sensitive to the particularities of the model and/or the *ad hoc* assumptions in particular studies. In contrast, a coordinated multi-model comparison like this puts the robustness (or lack thereof) of results in sharper focus.

Our tax trajectories span a range of typical proposals, but one should not construe our study as an analysis of any particular legislation. Several important differences between the stylized scenarios in this study and legislation arise. Many bills would cover a broader set of GHGs and include border carbon adjustments for energy-intensive trade-exposed industries.^7^ Some would include new spending, such as on infrastructure or assistance for coal workers and coal reliant communities,^8^ and some might limit state-level policies or roll back renewable energy subsidies, which we hold constant. Nonetheless, it is instructive to compare different policy scenarios that hold all features constant save for a particular design option of interest.

## 3. Results

This section reviews the headline results for the core scenarios; Sec. 4 below discusses how and why model results differ. The analysis will focus on the timeframe from 2020 to 2040. Although the models run past 2040, uncertainty amplifies so considerably over the longer term that the insights from the scenarios erode. We begin with the environmental performance of the policies and the way the policies shape the future of the energy sector. We then review the revenue estimations and the economic outcomes, with special attention to gross domestic product (GDP), its components, and welfare. For a nontechnical discussion of these and other results from the EMF 32 study, paying special attention to their policy relevance, see Barron *et al.* (this issue).

### 3.1. Emissions

**Figure 2.**
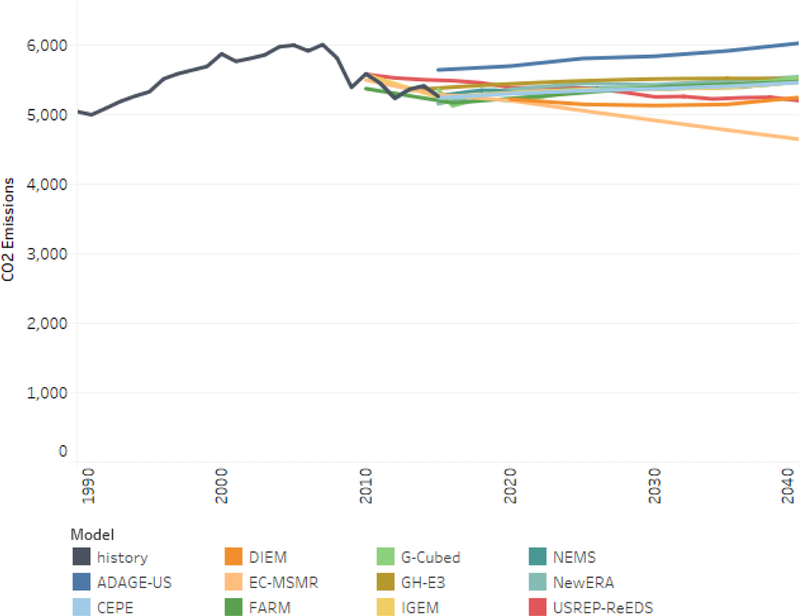
Reference case CO_2_ emissions from fossil fuel consumption.

We measure the emissions performance of a carbon tax relative to what would happen without the policy, i.e., the reference scenario. Figure 2 shows actual U.S. carbon dioxide emissions from fossil fuel consumption from 1990 to 2015 (in black) as reported by the EIA, followed by each model’s reference case emissions from 2015 to 2040, differentiated by color. The vertical axis is in units of millions of metric tons of CO_2_ (MMTCO_2_). As noted earlier, modeling teams calibrated their reference scenarios as closely as possible to the “No Clean Power Plan” trajectory from EIA’s April 2016 AEO. As a result of this harmonization, most of the trajectories are similar and show very low growth in emissions over the next 25 years. A few curves stand out. ADAGE-US has higher initial emissions but a similar growth rate to most of the other models. EC-MSMR, in contrast, starts in the middle of the group but shows a pronounced decline in emissions. Finally, USREP-ReEDS shows a modest decline through 2040, and DIEM shows an initial decline followed by a subsequent rise in emissions.

**Figure 3.**
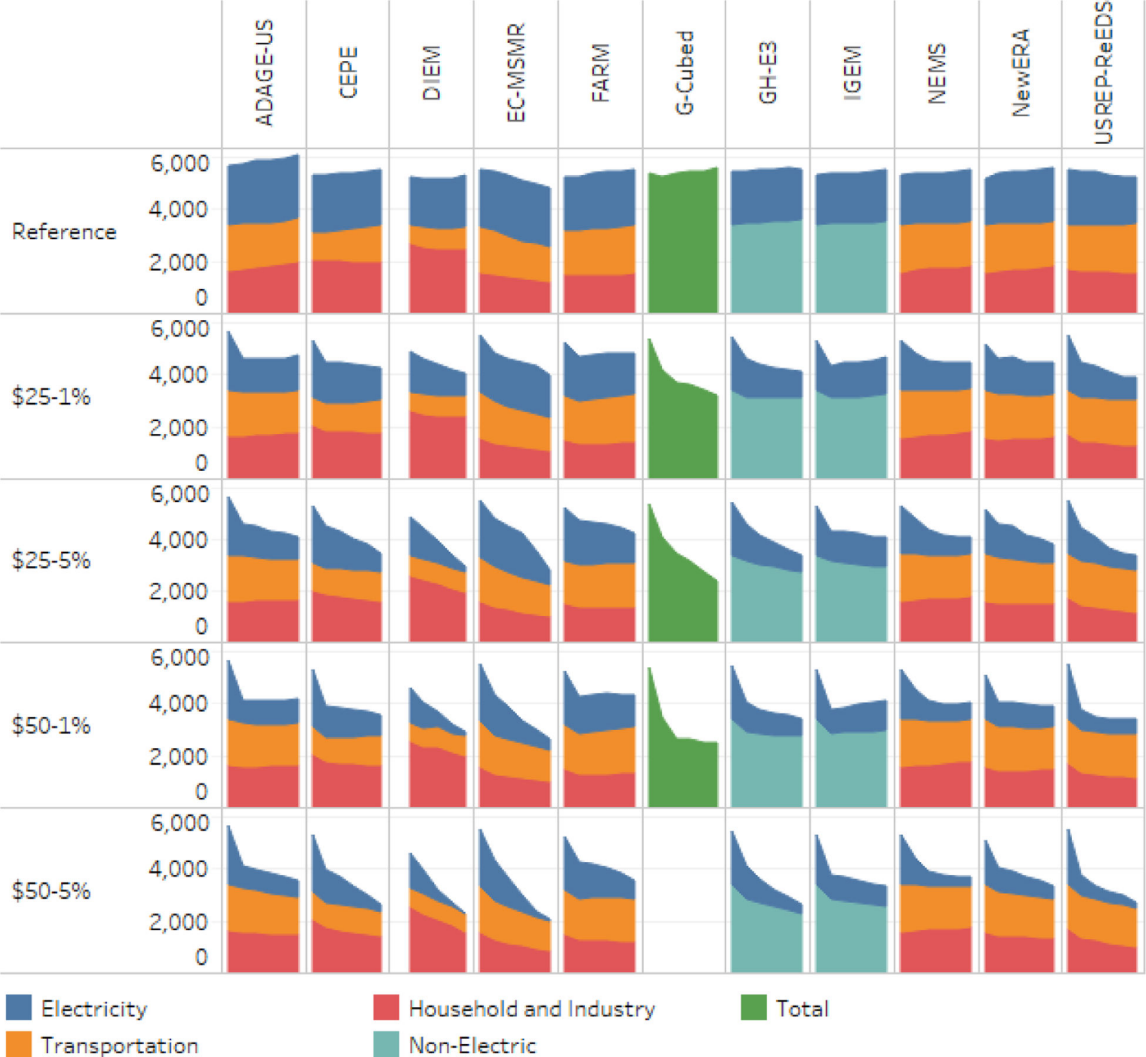
CO_2_ emissions in MMTCO_2_ by sector with household rebates, 2015–2040.

Figure 3 shows CO_2_ emissions by sector for each model under the reference case (first row) and each of the core carbon tax scenarios that rebates revenues to households. Each column in the figure reports the results of a particular model. The vertical axis in each panel is scaled in MMTCO_2_, and each horizontal axis ranges from 2015 to 2040. For simplicity, we focus in this section on the household rebate scenarios. The emissions outcomes for the other revenue recycling scenarios are very similar, as we discuss subsequently. Where data are available, the sectors shown are: (1) electricity generation, at the top in blue; (2) transportation, in orange; and (3) all other sources, in red. Some models provided less detail; GH-E3 and IGEM reported only electric and nonelectric emissions, whereas G-Cubed reported only total CO_2_ emissions.

The reference case emissions in the electricity sector (in blue in the top row) are generally consistent across models and through time. Some small differences arise in ADAGE-US, which has slightly larger electric sector emissions than the other models, and USREP-ReEDS, which projects emissions that fall slightly through 2040.

In contrast, sharp differences arise across models in the sectoral composition of emissions outside the electric sector. DIEM and NewERA project relatively low emissions from transportation and relatively high emissions from all other sources, whereas C-MSMR, FARM, NEMS, and ADAGE-US project relatively high emissions from transportation and relatively low emissions from other sources. As will be discussed in more detail below, these differences reflect differences in the accounting conventions used by the models to allocate emissions from petroleum consumption to different sectors. Models with low transportation emissions use a narrow definition; hence, a significant portion of residual emissions for those models is ultimately attributable to transportation.

Subsequent rows in Fig. 3 show how emissions respond to different carbon taxes. In all models, a carbon tax causes a substantial initial decline in electric sector emissions when it is implemented in 2020. As would be expected, the initial decline is largely driven by the initial tax rate: the two $25 policies show similar impacts in 2020, as do the two $50 policies, and the $50 policies show larger initial impacts than the $25 runs.

**Figure 4.**
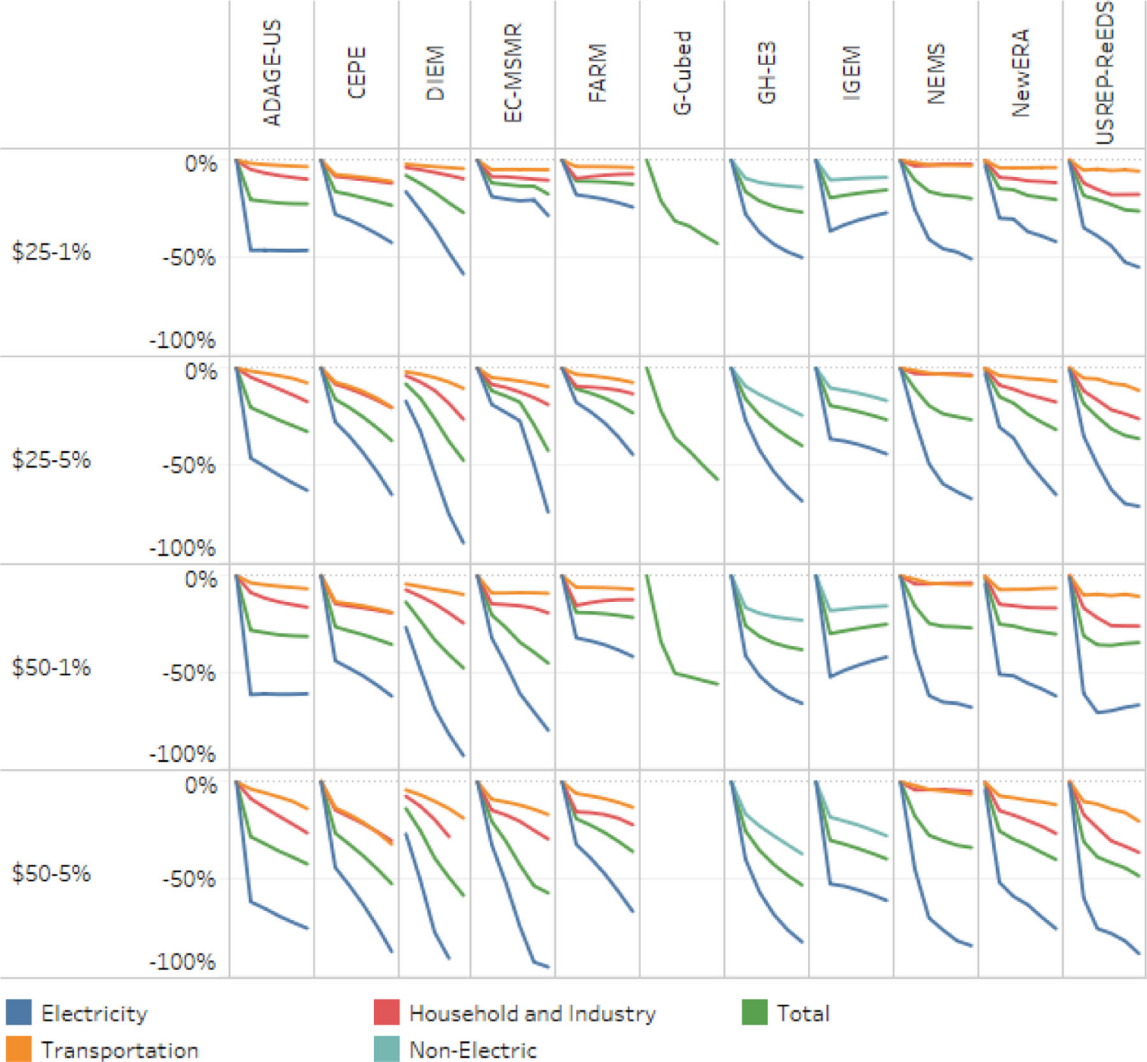
Percent change in emissions relative to baseline, by sector with household rebates, 2015–2040.

In general, we find that even the most ambitious carbon taxes have little impact outside of electricity generation; changes from the reference case in both transportation and other emissions are small. Figure 4 shows this more clearly by reporting each model’s sectoral change in emissions measured as a percent of that sectors’ reference value, again from 2020 to 2040. All of the models show substantial percentage changes in electric sector emissions and smaller percentage changes in other sectors. This illustrates the limitation in some models of inadequate representation of alternative vehicles (such as plug-in electric vehicles) and biofuels, along with the limitation that models cannot anticipate potentially profound future technological developments in the transportation sector.

**Figure 5.**
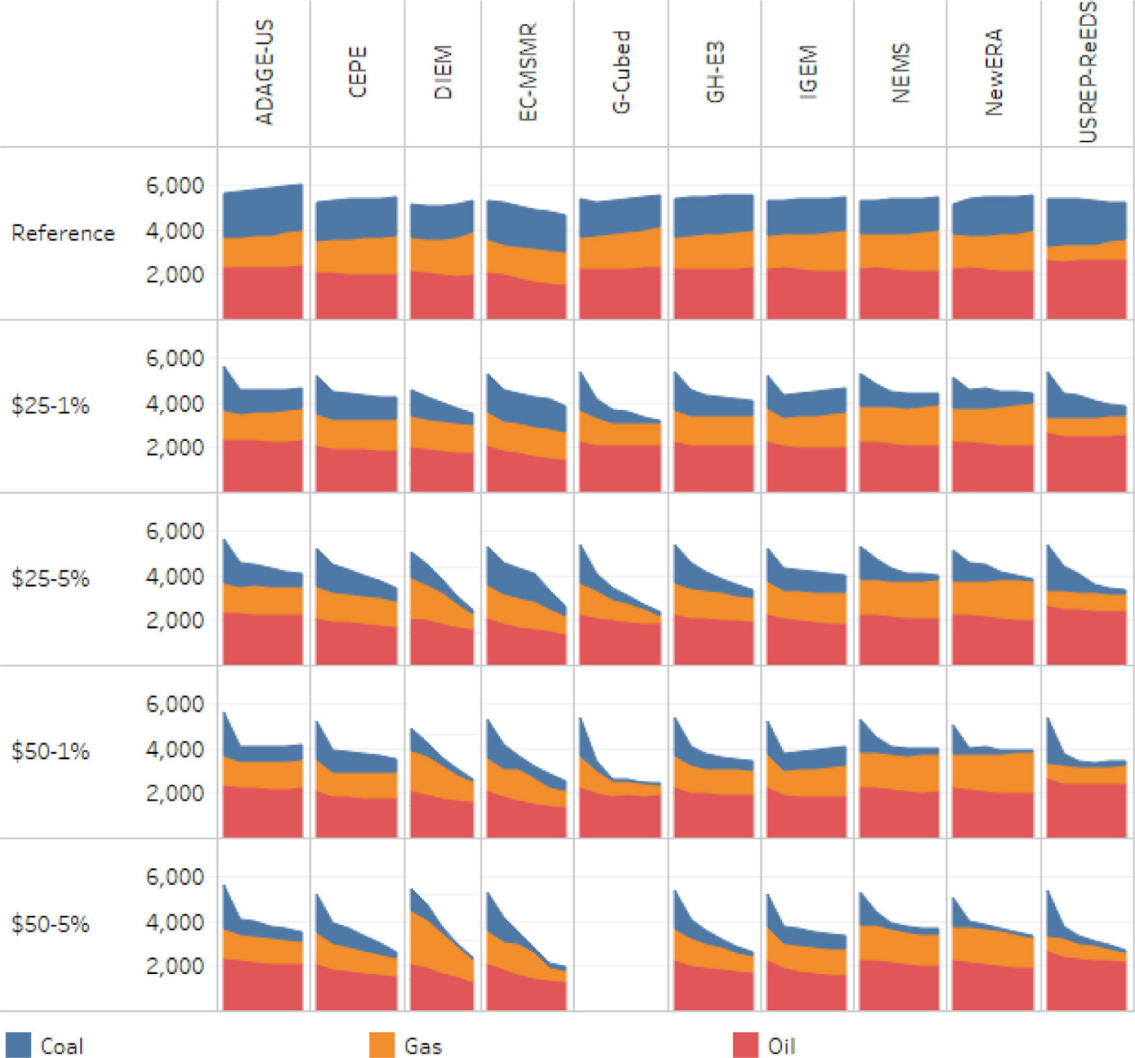
CO_2_ emissions by fuel, with household rebates 2015–2040.

**Figure 6.**
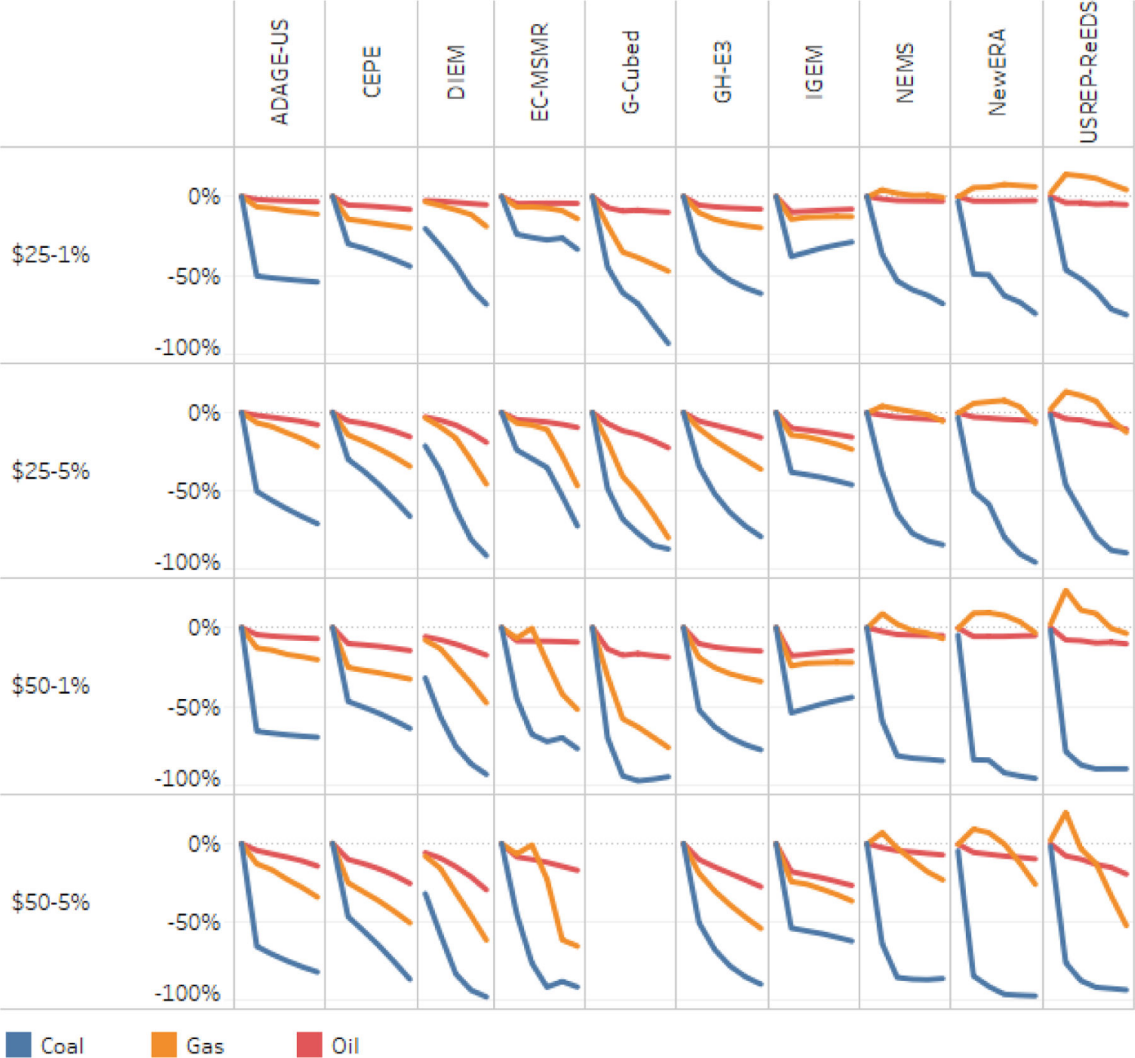
Percent change in emissions by fuel from reference, with household rebates 2015–2040.

Figures 5 and 6 provide a different view of emissions by allocating them to fossil fuels (coal, natural gas, and oil) rather than end-use sectors. This approach is more straightforward, and as a result the models’ findings by fuel are more consistent than by sector. Consistency across the models in emissions from oil reinforces how differences in emissions attributed to transportation largely arise due to differences in accounting conventions. Models with low transportation emissions are allocating a significant fraction of emissions from oil consumption to the residual category rather than transportation. Models with atypical reference results include DIEM, which has sharply higher emissions than other models, EC-MSMR, which as noted above has falling emissions in the reference, and USREP-ReEDS, which has unusually low emissions from natural gas and unusually high emissions from oil.

Turning to the carbon tax results in Fig. 5, the models consistently show that declines in coal-related emissions disproportionately account for overall CO_2_ abatement. The impact begins immediately in 2020 and generally grows over time as the tax rises. The models are also very consistent in showing proportionately less abatement from oil consumption, consistent with the inelastic response in the transport sector shown in Fig. 4. Impacts on emissions from natural gas differ the most across the models.

Figure 6 further illustrates the similarities and differences across the models by showing percentage changes from the reference case in emissions by fuel. Coal emissions fall most dramatically in most of the models, along with small changes in oilemissions, and a mix of impacts on gas emissions. A few models and scenarios report an increase in natural gas-related emissions, suggesting that natural gas is picking up some of the market share lost by coal.

**Figure 7.**
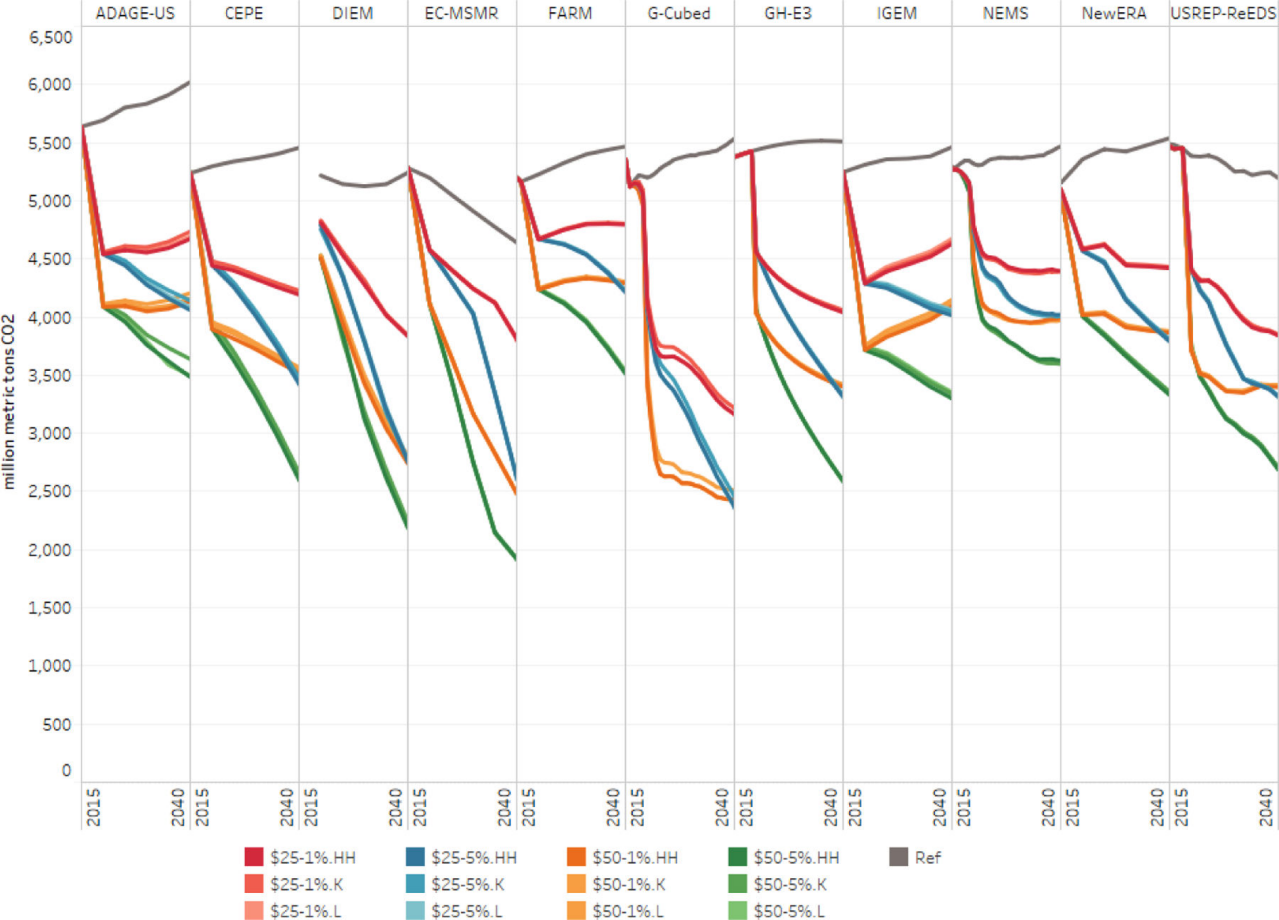
CO_2_ emissions in reference and core carbon tax scenarios, 2015–2040.

Now let us consider the emissions outcomes across the range of recycling options. Figure 7 shows emissions for the reference case and the core carbon tax scenarios by model. In each panel, the reference case is the top curve (in black). The other curves are grouped and colored first by stringency and then by recycling option. For example, the first group of runs, which are different tones of red, has a carbon tax starting at $25 per ton and rising at 1% per year. Within the group, the curves appear in the following order (and with decreasing darkness in the figure): household rebates (*HH*), capital tax recycling (*K*), and then labor tax recycling (*L*). The vertical axis is in MMTCO_2_, and the horizontal axis spans the year 2015 to 2040.

Figure 7 shows that, for a given carbon tax trajectory, different revenue recycling options produce very similar emissions reductions. For example, the light orange curves deviate very little from the darker orange curves. Slight differences arise in some models, such as ADAGE, in which recycling via a capital tax reduction results in slightly greater economic activity — and higher emissions — relative to the scenario with household rebates. In all cases, the differences are modest but more pronounced in more ambitious carbon tax trajectories.

One significant difference across the models is the long-term impact of the tax policies that grow at 1% per year (the red and orange groups). For about half the models, a 1% growth rate is more than enough to offset the rate of emissions growth built into the reference case; 2040 emissions levels are below the 2020 levels. However, for several models that is not the case; FARM, IGEM, and ADAGE-US all show that underlying emissions growth is not fully offset by growth of the tax, and 2040 emissions end up higher than in 2020. To be sure, emissions in all of the policy scenarios are well below baseline levels, but to ensure that emissions continue to fall as the economy grows, these results suggest that a rate of increase over 1% per year over inflation is necessary. Emissions fall consistently through 2040 in all of the scenarios that increase the tax rate at 5% over inflation.

Consistent with Fig. 4, long-term emissions outcomes are roughly similar in several of the models; FARM, IGEM, NEMS, NewERA, and ADAGE-US all have comparable emissions levels in 2040, although for ADAGE the 2040 level represents a larger decline from its growing baseline emissions. Significant differences between the models arise at the onset of the policy in 2020 and along the transition path. CEPE, DIEM, G-Cubed, and USREP-ReEDS generally show larger impacts, with the largest occuring in G-Cubed. EC-MSMR differs from the others in having the smallest impacts relative to the reference case, which for EC-MSMR is declining sharply through 2040.

### 3.2. Energy sector outcomes

**Figure 8.**
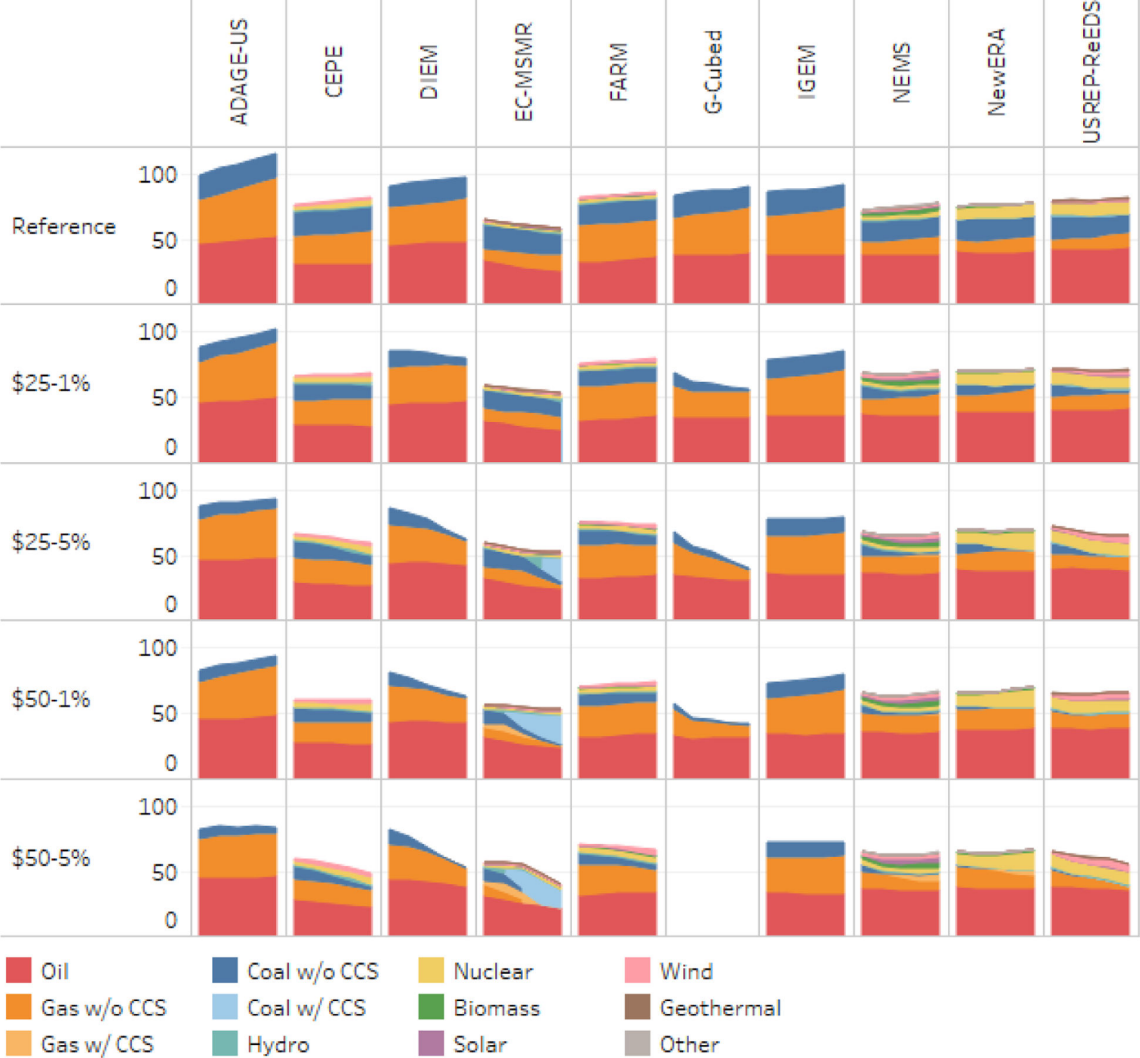
Primary energy consumption, with household rebates 2020–2040, exajoules per year.

As detailed in Macaluso *et al.* (this issue), the EMF 32 carbon tax scenarios produce significant shifts in the U.S. energy sector. One result of interest we highlight here is the extent to which CO_2_ emissions fall through a substitution to lower carbon fuels or by a reduction in energy consumption, such as via greater energy efficiency. Figure 8 illustrates these outcomes by reporting primary energy consumption by fuel, expressed in exajoules (EJ, or 10^18^ J).^9^ Primary energy is a measure that sums all of the domestic consumption of energy and adds net energy imports. It includes the consumption and net imports of all fossil fuels and other sources of energy, such as nuclear power and renewable electricity. Consistent with the AEO values used for calibration, Fig. 8 shows that most of the models project a baseline of gradually rising energy consumption (first row), but the compositions of energy sources vary somewhat across models. EC-MSMR projects falling energy consumption from oil. Other models with atypical reference case projections include DIEM and EC-MSMR, which project higher oil consumption than most models, USREP-ReEDS, which projects substantially lower gas consumption, and ADAGE-US, which has higher long-term energy consumption than the other models, largely due to a significant expansion in natural gas.

The second row of Fig. 8, the $25 rising at 1% carbon tax scenario, shows how responsive total energy consumption is to a carbon price. The most responsive models are CEPE, DIEM, G-Cubed, and USREP-ReEDS. Even with a modest tax trajectory, they project an absolute decline in energy consumption from 2020 to 2040 relative to a stable or growing baseline. Moving down the rows, as the tax becomes more stringent, the declines in energy consumption become more pronounced. Under the most stringent policy, $50 rising at 5%, all models show declining energy consumption over the period.

**Figure 9.**
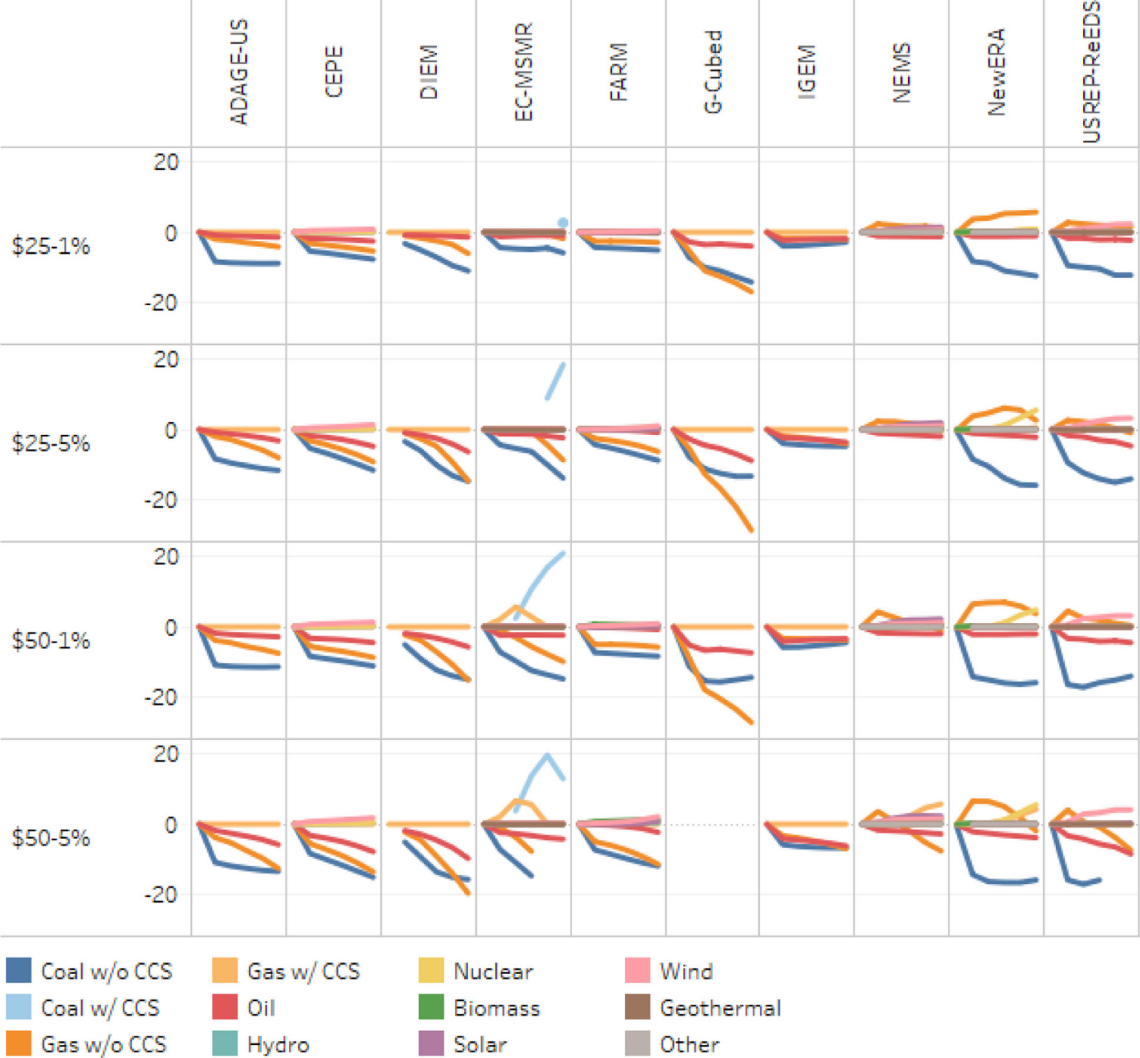
Change in primary energy consumption relative to reference (EJ per year), with household rebates, 2015–2040.

Absolute declines in consumption of energy by fuel (relative to reference) from 2015 to 2040 appear in Fig. 9. As expected from the emissions results, coal without CCS declines substantially in almost all models, even for the least stringent policy. EC-MSMR projects a strong increase in coal with CCS, but in the most stringent scenario that declines in the out-years. Where the models differ most prominently is the impact of the tax on gas. Gas consumption declines in one group of models (CEPE, DIEM, EC-MSMR, FARM, G-Cubed, IGEM, and ADAGE-US) but increases or is unaffected in all but the most stringent cases in the other group (NEMS, NewERA, and USREP-ReEDS). G-Cubed reports the largest drop in gas consumption and NEMS shows the smallest. The difference between the two sets of results stems in part from the approach used by the models for representing electricity generation: those with relatively large impacts use nested cost or consumption functions, whereas those showing small impacts use detailed linear programming (LP) models.

Among models that report the electric sector in detail, CEPE, FARM, NEMS, and USREP-ReEDS show modest increases in wind energy. NEMS also reports significant additional solar energy, particularly under more stringent policies. NewERA also reports a considerable expansion of nuclear power after about 2025 under the middle and higher tax scenarios. Comparing Fig. 9 to the earlier figure on emissions reductions shows that a key source of the differences between models in emissions is the underlying difference in the impact of a carbon tax on natural gas.

### 3.3. Revenue

**Figure 10.**
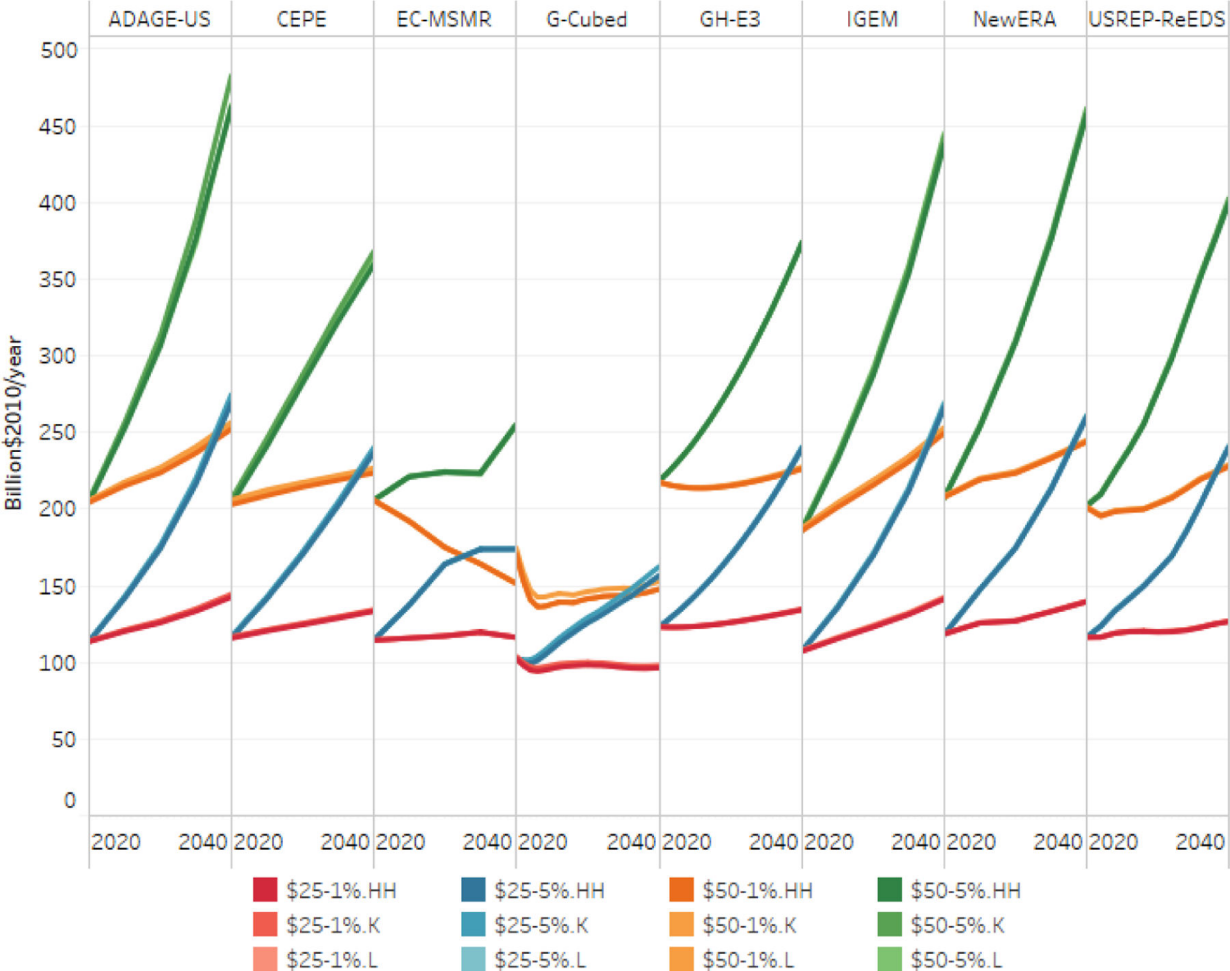
Gross annual real carbon revenue by tax trajectory and recycling option, 2020–2040.

We have seen so far that all of the carbon tax policies reduce emissions, in some cases very substantially. Thus, the question arises how much the annual increase in the real carbon tax rate offsets the decline emissions to affect revenue. Figure 10 reports the revenue path from 2020 to 2040 for the core policy scenarios. Annual revenues for the paths that begin at $25 start at about $100 billion per year or slightly above that. Tax paths that start at $50 per ton raise slightly less than double their $25 counterparts owing to their greater impact on emissions, as shown in Fig. 9. In general, with the exception of the EC-MSMR model, the revenue paths for the tax trajectories that rise at 1% over inflation are stable or slightly increasing in real terms. The tax paths that rise at 5% over inflation produce sharply increasing revenues through 2040. As a share of GDP (see supplementary figures), revenues decline in the 1% scenarios and increase or remain constant in the 5% scenarios. Not surprisingly, models with relatively lower emissions abatement report the highest revenue streams.

**Figure 11.**
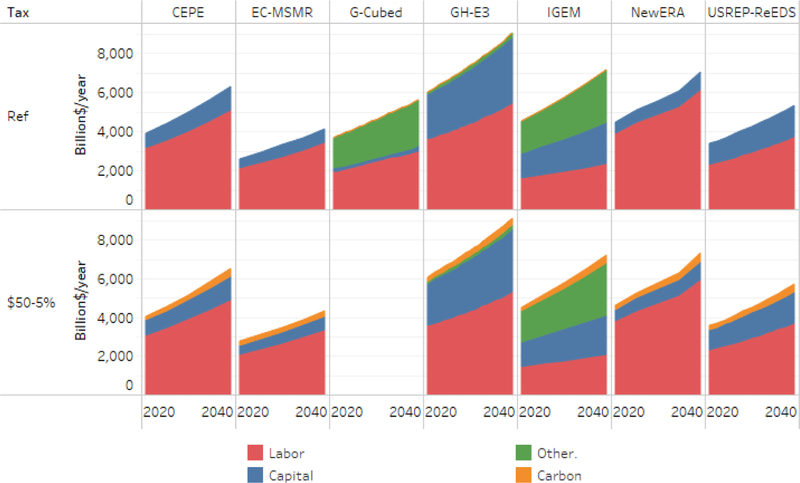
Gross federal revenue composition under reference and household recycling.

To illustrate how big a role a carbon tax would play in the overall federal revenue system, Fig. 11 shows the composition of total federal revenue in the reference case (first row) and the most ambitious core carbon tax scenario (second row). Owing to the different ways the models represent different tax instruments, the reference case varies significantly across models in both the relative shares of labor, capital, and other taxes and in the total revenue. However, the relevant insight comes from the second row, which includes the carbon tax in the mix. In every model, the carbon tax introduces a small but nontrivial slice of new net revenue.

A carbon tax can reduce overall economic activity and shift the returns to capital and labor. As CBO (2009) notes, when excise taxes, customs duties, and other types of “indirect” taxes are imposed on goods and services, they tend to reduce income for workers or business owners in the taxed industry and for others throughout the economy. That means that while a carbon tax raises revenue, its effects can lower revenues from other taxes, especially corporate and personal income and payroll taxes. Accordingly, the Joint Committee on Taxation (JCT) subtracts an offset, or “haircut”, to its estimate of the net revenue from carbon tax.^10^ For example, the JCT reduces its estimate of the net revenue from excise taxes by subtracting about 25% from the expected gross revenue. JCT gradually increases the offset rate from time to time to account for forecasted income growth, which increases the average marginal individual income tax rate.

EMF 32 modeling results illustrate the differences between the gross revenue from a carbon tax and the amount of money available to recycle. In our scenarios, we hold government spending and the federal budget deficit constant relative to baseline. The household rebate scenarios provide equal rebates to all such that the total rebated revenue holds the federal deficit unchanged relative to baseline. In the tax swap scenarios, we calculate the change in the capital or labor income tax rate (in percentage points) achievable in each period, using the recycled revenue to hold the deficit constant relative to baseline.

**Figure 12.**
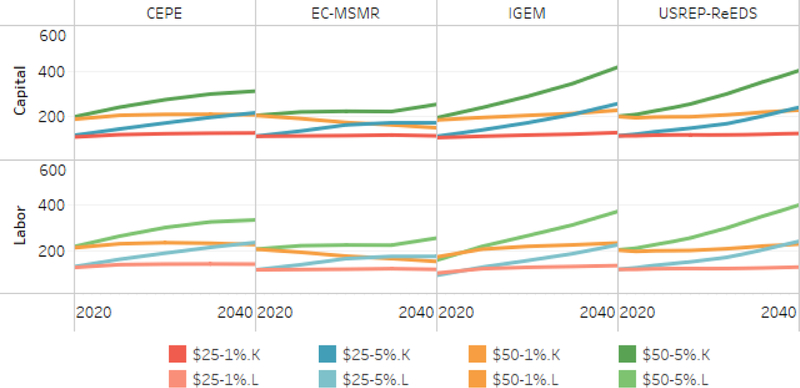
Revenue recycled to cuts in capital and labor tax rates (billion $/year).

Figure 12 reports the revenues that fund tax cuts in the capital and labor revenue recycling strategies for a subset of models reporting this information. Due to the changes in revenues from other taxes (i.e., the “haircut”), the revenue available to offset capital and labor is somewhat less than the revenue collected. In EC-MSMR and USREP-ReEDS, the revenue recycled is the same for both the capital and labor scenarios. CEPE recycles about 10% less in the labor tax recycling scenario; IGEM recycles about 5% more in the capital tax recycling scenario though this varies somewhat over time.

Our household rebate scenarios allow us to compute something akin to the offset, or haircut, that JCT imposes in its revenue score, recognizing that our scenarios do not necessarily hold the same factors constant relative to the reference case that JCT does. We count the rebates as direct spending, not a tax expenditure, and the scenarios specify that the total rebates must be scaled so that government deficits are unchanged relative to baseline. That means that total government revenue goes up with the carbon tax, but not as much as the gross revenue from the carbon excise tax, given the effects on other revenue instruments.

Table 5 reports the proportion by which the increase in total revenue from reference to policy differs from the gross receipts from the carbon tax. For example, in the $25–1% scenario, total rebates to households range across the five models from 3% to 40% lower than gross carbon tax receipts. These differences are driven by several underlying differences in the models. Labor supply elasticities can be particularly important because they affect how labor income tax revenue responds to the decrease in the real wage prompted by higher overall price levels. The size and scope of pre-existing taxes in the models matter as well. For example, some models include a more expansive or detailed set of revenue instruments, such as state and local taxes. Other than for ADAGE-US, we see no systematic relationship between the starting carbon tax rate and the magnitude of the haircut. Scenarios in which the real carbon tax grows more slowly generally report slightly lower offsets for a given model. Some of the models, such as EC-MSMR and G-Cubed, project offsets very similar to those employed by the Joint Committee on Taxation in scoring excise tax legislation.^11^

### 3.4. Macroeconomic outcomes

**Figure 13.**
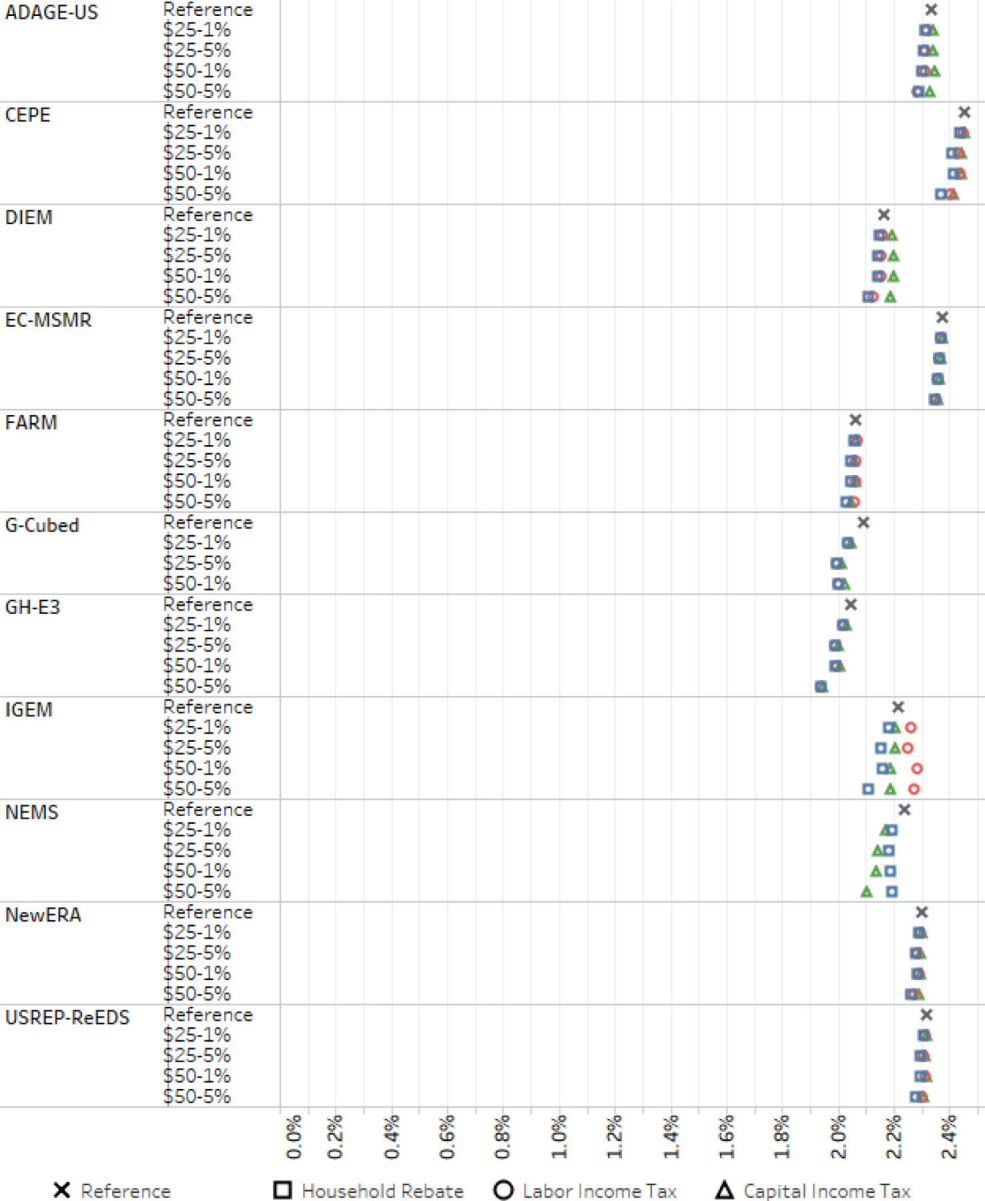
Average annual percent growth in GDP from 2015 to 2040 under reference and core scenarios.

Here we turn to the macroeconomic outcomes of the carbon tax policies. Before presenting any of the macroeconomic outcomes, it is worth noting again that none of the models in this study include any of the impacts of climate change in the reference cases or the benefits of reduced GHG emissions in the policy cases. The results of this study are useful as a cost-effectiveness analysis comparing different policies, but a full consideration of the policies presented here would also require a consideration of the benefits, which is outside of the scope of EMF 32. Figure 13 shows average GDP growth rates from 2015 to 2040 in the different scenarios. Reference average growth rates, indicated by the marker *x* in the figure, range across models between 2.0% and 2.5% points. Growth rates in the policy scenarios are slightly different and in some cases vary by the revenue recycling scenario.

The most important takeaway from this figure is that in every policy scenario, in every model, the U.S. economy continues to grow at or near its long-term average baseline rate, deviating from reference growth by no more than about 0.1% points. We find robust evidence that even the most ambitious carbon tax is consistent with long-term positive economic growth, near baseline rates, not even counting the growth benefits of a less-disrupted climate or lower ambient air pollution. A few of the models show a very small effect of incremental stringency of the climate policy on average growth rates (EC-MSMR, FARM, and NewERA); others (CEPE, DIEM, G-Cubed, GH-E3, and IGEM, and GH-E3) show more distinct decreases in average growth with greater stringency.

A number of models (including EC-MSMR, GH-E3, and NewERA) demonstrate that, for any given carbon tax trajectory, different revenue recycling approaches result in nearly imperceptible differences in average growth rates. In models in which differences do arise, most (ADAGE-US, CEPE, DIEM, FARM, and USREP-ReEDS) show slightly higher growth rates in the capital tax swap scenarios than the other revenue scenarios. In DIEM and ADAGE-US, capital tax recycling scenarios demonstrate higher-than-baseline growth.

IGEM and NEMS are notably different from the other models. IGEM exhibits the widest range of outcomes by revenue recycling scenario. It projects about 0.1% points higher-than-baseline average growth with labor income tax recycling but about the same magnitude of lower-than-baseline growth with household rebates. NEMS is the only model in which capital tax recycling leads to lower growth rates than household rebates. Notably, it is also the only macroeconomic model; all others are CGE models. The household rebates in NEMS stimulate consumption, whereas the reduction in the capital tax rate exhibits both lower consumption and investment relative to household rebates. See Arora *et al.* (this issue) for further analysis of NEMS behavior.

**Figure 14.**
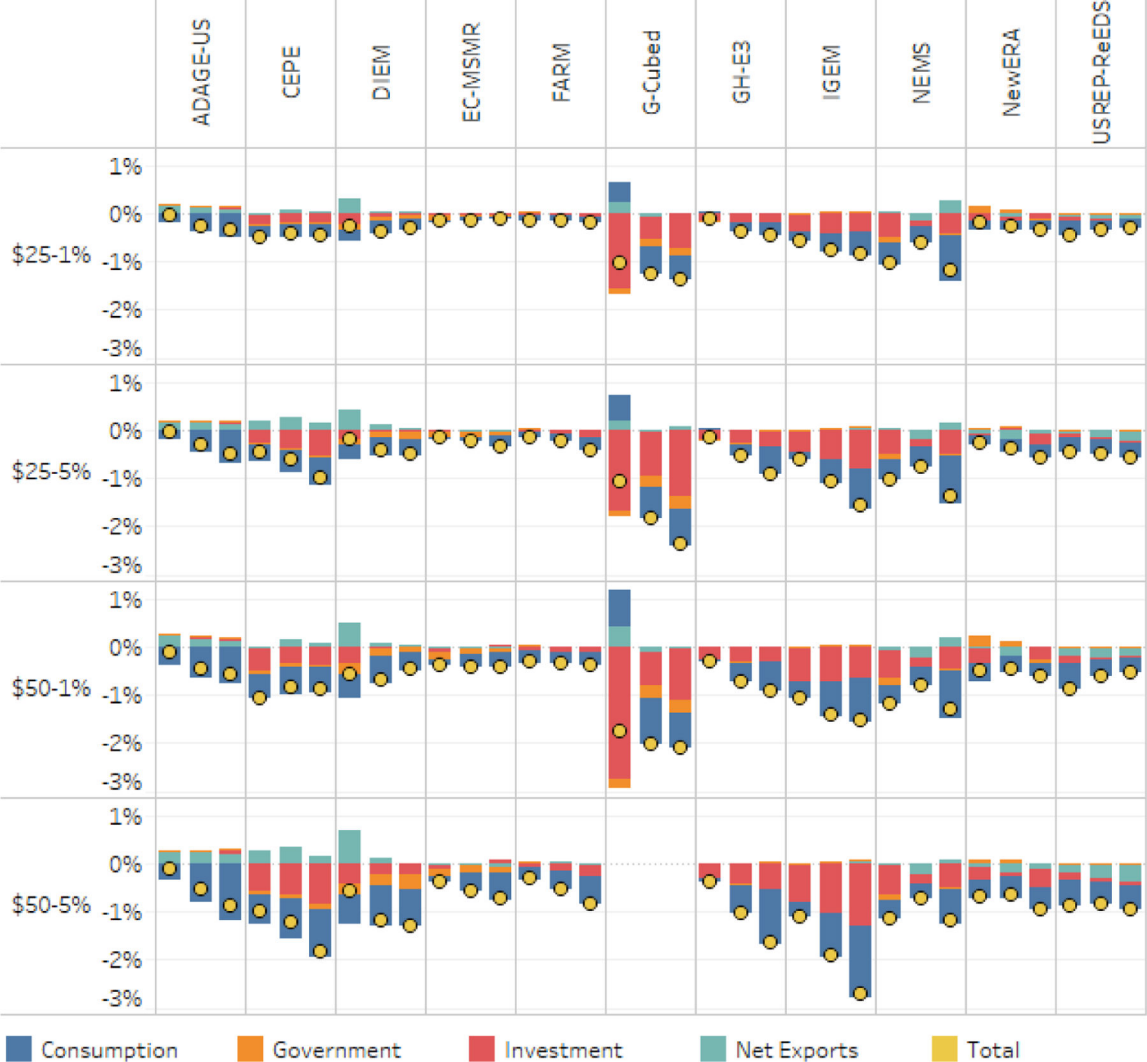
Change in GDP and its components as a percent of reference levels of GDP in 2020, 2030, and 2040, with household rebates.

Figure 14 reports changes in overall GDP and its components (consumption, investment, government spending, and net exports) as a percent of reference levels of GDP in 2020, 2030, and 2040. As in some cases components of GDP increase relative to reference, we denote the net effect on total GDP with yellow dots. All of the scenarios use carbon tax revenue for household rebates (see supplementary materials for labor and capital tax swap results). Consistent with the results in Fig. 13, we see that all of these scenarios result in negative net effects on GDP relative to reference, in most instances with the negative effects accumulating over time. Some models, such as NEMS and DIEM, report nonmonotonic effects on GDP.

Several of the models show a fairly even impact across the major components of GDP with each decreasing proportional to the overall GDP impact. The exceptions are some (but not all) of the perfect foresight models.^12^ These few models tend to show increases in consumption in the short run as the long-term implications of the policy are anticipated. Some (but not all) of the perfect foresight models show disproportional reductions in investment, which appears to explain the larger overall GDP impacts in results from these models. The G-Cubed model shows a larger impact and different temporal pattern of results for the components of GDP compared with the other models, see McKibbin *et al.* (this issue) for more information of these results.

For the $50–5% scenario, Fig. 14 suggests that the magnitude of impact on GDP is centered around −1.0%, with a few models showing impacts as large as −2% to −3%, and some as low as −0.5% to +0.75%. All models that simulated both the $50–1% and the $50–5% scenarios showed lower impacts for the former than the latter, centered around a fall of about −0.75% with some as large as −1.5% to 2.0% and some as low as ~ −0.25%. For the $25–1% and $25–5% many of the models showed negligible effects on GDP (< −0.2%) but a few models showed impacts greater than −1.0% or 1.5%.

Reviewing the results in Fig. 14 for 2040, we find that only a couple of the model/ scenario runs report GDP lower than about 2% below baseline. This means that with the least efficient revenue use of our core scenarios, the U.S. economy loses at most about the equivalent of about 1 year of economic growth through 2040. Most outcomes are less than half of that.

**Figure 15.**
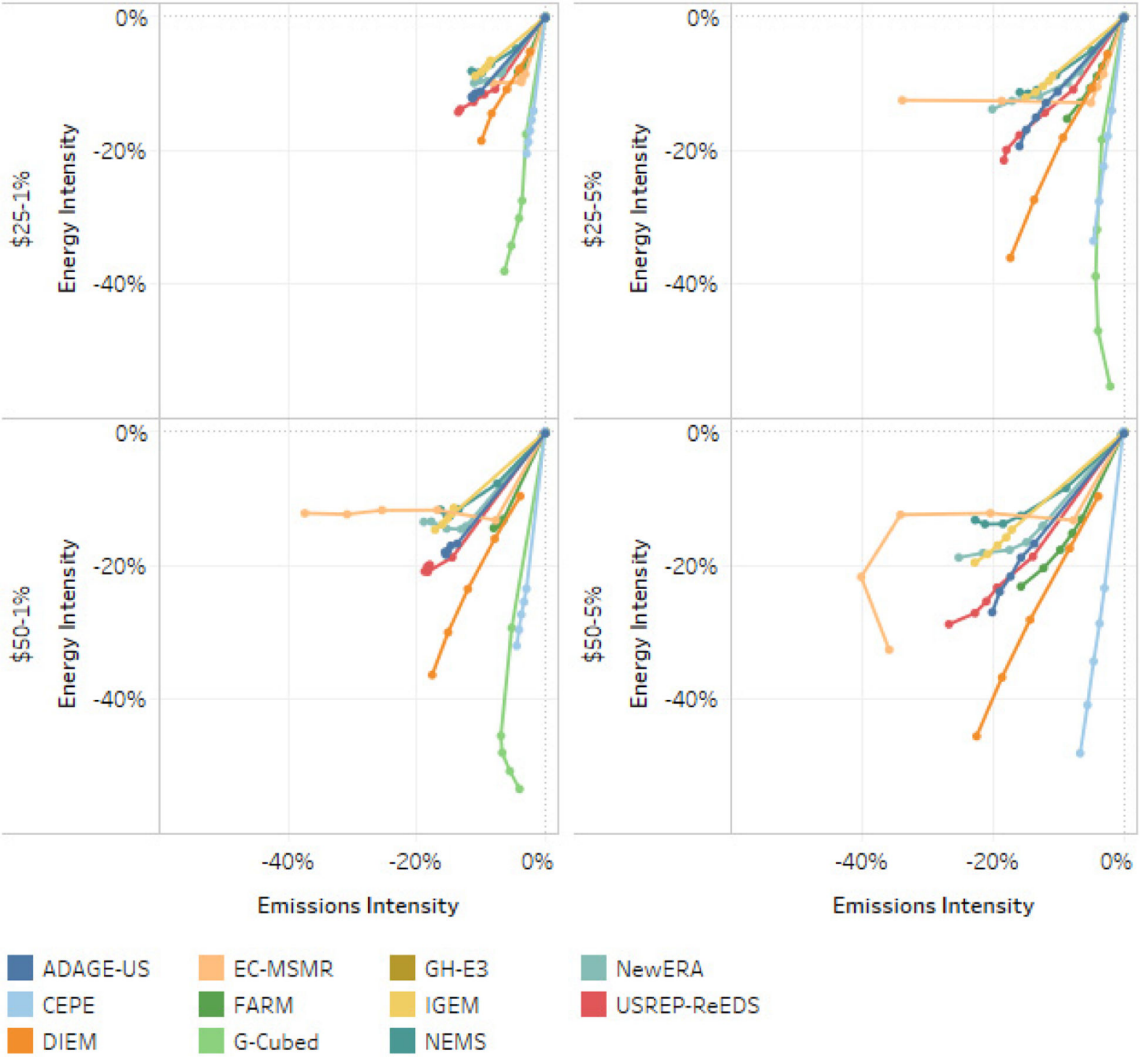
Primary energy intensity (primary energy/GDP) and emissions intensity (emissions/primary energy) percent changes relative to reference, 2015–2040.

Figure 15 illustrates how models vary in the way their projections become less energy- and emissions-intensive as the carbon price ramps up. The curves for each model start in the upper left corners of the panel, with the origin scaled to each model’s 2015 levels of energy (in EJ) per dollar of GDP and CO_2_ emissions (in million metric tons of CO_2_) per unit of energy (in EJ). Each consecutive point on the curves represents the percentage change relative to reference of the following period’s intensity measures. The figure shows that some models, such as G-Cubed and CEPE (in light green and light blue, respectively), project greater relative reduction in energy intensity than emissions intensity. That is, they suggest that the policies disproportionately reduce energy use per unit GDP, relative to baseline, more than emissions per unit of energy. This can arise both from strong energy conservation and efficiency responses as well as structural shifts to less energy-intensive production and consumption activities. Other models, such as IGEM and NEMS, show greater relative reductions in emissions per unit of energy, suggesting relatively more shifting of energy production to low-carbon alternatives. Others, such as USREP-ReEDS, follow closer to the diagonal, indicating similar proportional reductions in each measure. EC-MSMR, particularly for the $25–5% and $50–1% scenarios, illustrates the adoption of a low-carbon backstop technology (coal with CCS). Emissions intensity falls with negligible change in energy intensity. See Macaluso *et al.* (this issue) for a more extensive decomposition of emissions and energy reductions.

### 3.5. Welfare effects

So far, we have seen robust evidence that the revenue recycling option is unlikely to affect a carbon tax’s emissions reductions, energy sector outcomes, or gross revenue. With macroeconomic outcomes such as GDP, consistent with earlier research, we have seen that the revenue recycling option is more important. Here we present another way to look at the overall social costs of the abatement policy using an equivalent variation (EV) measure of welfare losses relative to reference over the period 2020–2040. An EV is a measure of the change in economic welfare associated with a change in prices.^13^ It reflects the change in income necessary to get to the new level of utility at the original prices. Positive numbers in the figure indicate welfare losses to U.S. households over the period 2020–2040 under the policy scenarios, relative to reference. Thus, the welfare effects take into account both the impacts of the carbon tax and how the revenue is used. To aggregate losses that occur in different years, we discount real future losses to 2020 using a 3% discount rate. As discussed in the limitations section, it is important to note that the welfare effects presented here do not take into account any of the impacts associated with climate change or the benefits of mitigating GHGs and conventional pollutants.

We report the cumulative losses in welfare from the carbon tax in two different ways: scaled by cumulative consumption and per ton of CO_2_ reduced relative to reference.^14^

**Figure 16.**
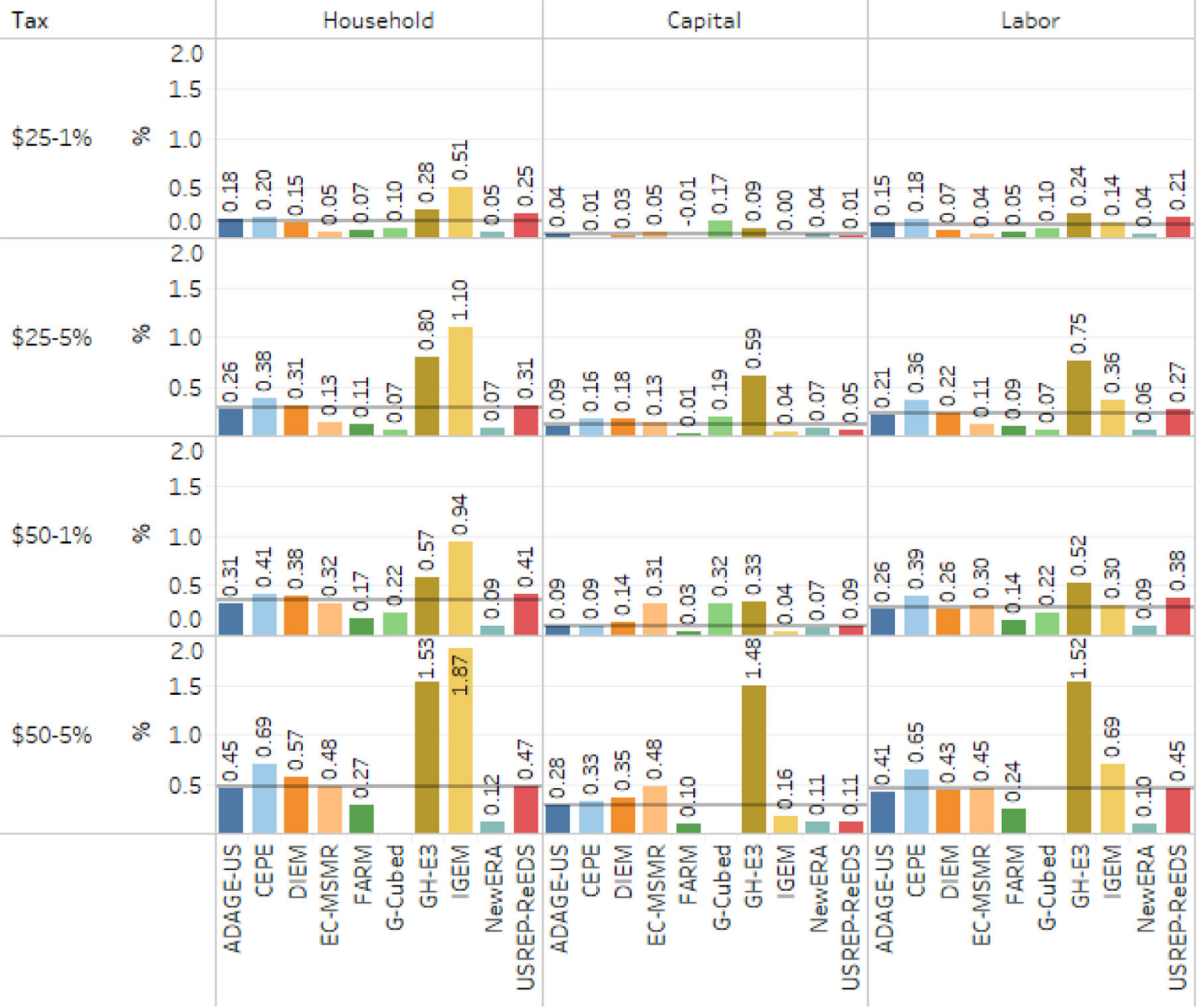
Cumulative welfare reduction (EV) as percent of cumulative consumption for core scenarios from 2020 to 2040, discounted at 3%. Median across models represented by straight line.

Figure 16 shows the EV as a percentage of cumulative consumption from 2020 to 2040. This puts aggregate EVs in context. For example, in the $25–5% scenario with capital income tax recycling, the cumulative EV loss ranges up to $2 trillion with a 3% discount rate (see supplementary materials Fig. S6). Here, we see that amounts to at most about 0.6% of cumulative consumption. The average across models for that scenario is about 0.15% of cumulative consumption.

**Figure 17.**
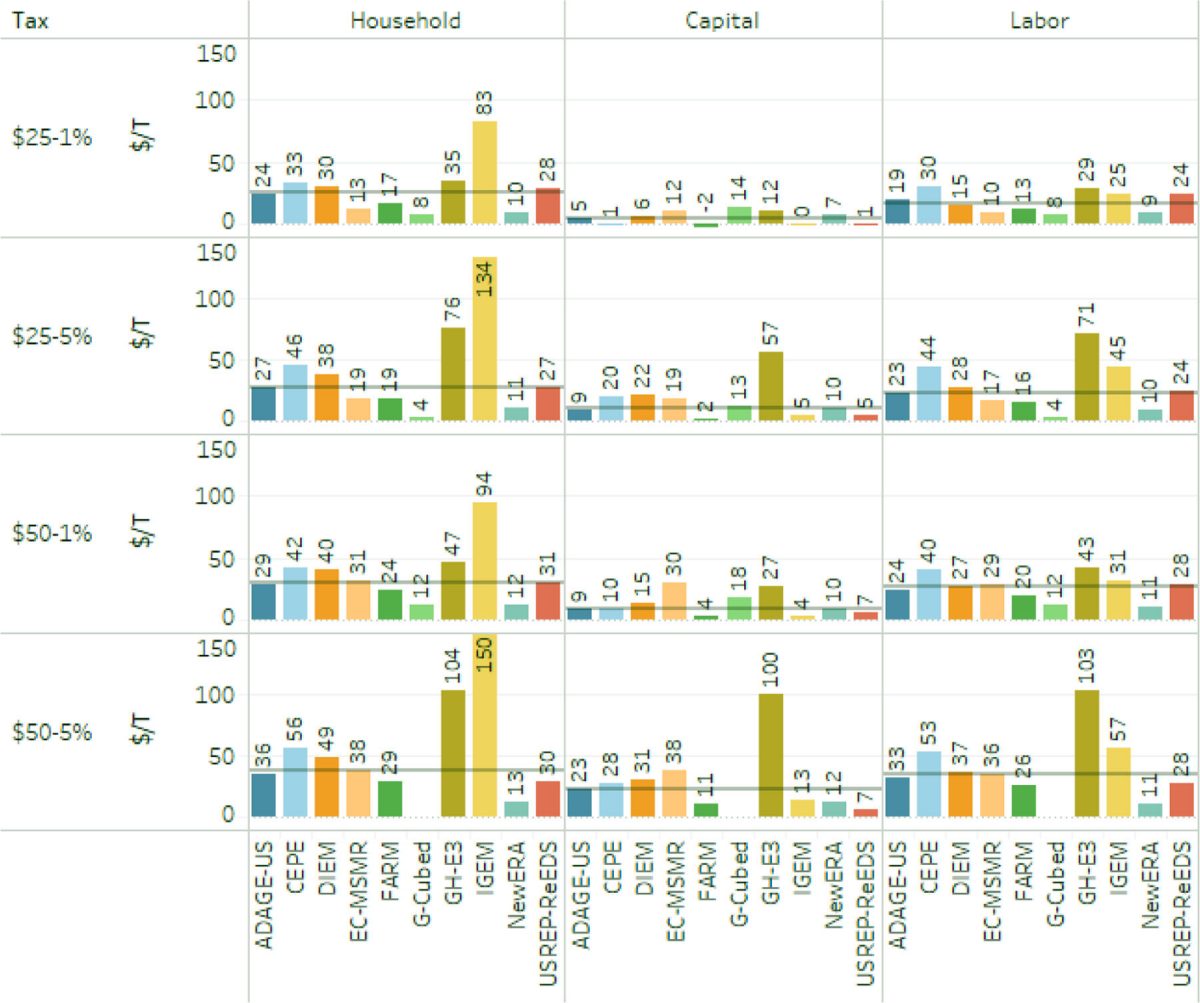
Cumulative welfare reduction per ton of CO_2_ abated, by tax scenario, recycling option, and model discounted at 3% over 2020–2040.

Because welfare impacts and emissions reductions both vary over time, a useful comparison is each model’s average EV per cumulative ton reduced (relative to reference) over the period from 2020 to 2040. The results appear in Fig. 17, which has one row for each core carbon tax, one column for each recycling mechanism, and separate bars for each model. Lump-sum recycling appears in the left column.

This metric is useful because it takes into account both abatement cost and emissions abated. Similar in magnitude to a carbon price, the carbon price is a marginal cost concept applying to the last ton abated in any year, whereas the NPV welfare cost per ton is an average across tons in any year and over time (discounted). Discounted at 3%, carbon price scenarios rising at 5% imply a slight increase in the discounted price over time, while scenarios with a 1% rise imply a slight decrease in the carbon price over time. As an average cost concept, we would expect the NPV welfare cost to be less than the average carbon price on first principles, but will vary depending on interactions with taxes and other distortions in the economy, due to growth and revenue effects.

### Welfare effects per ton of CO_2_ emissions — comparison across models.

Welfare costs per ton of emissions abated are generally highest when revenue is rebated to households and lowest when recycled by cutting capital taxes, with the exception of one model which showed capital tax recycling to be the highest cost option. The models with the highest cost per ton for the $25–1% carbon tax with rebates are IGEM and USREP-ReEDS, both of which have values above $50 per ton. In that same scenario, models with the lowest welfare loss include EC-MSMR, G-Cubed, and NewERA, all of which are on the order of $10 per ton or less. Moving to more stringent policies, such as $50–1% or $50–5%, raises the average welfare cost per ton in most models. That indicates that the corresponding implicit marginal abatement cost curves are upward sloping. An exception is USREP-ReEDS, in which the average welfare cost decreases.

### Welfare effects per ton of CO_2_ emissions — with household rebates.

For the $50–5% and 50–1% tax trajectories, welfare costs for 13 of the 19 runs ranged from about $24 to $56 per ton. Of the remaining sets of results, three were much higher (> $94) and three much lower ( < $13). For all the models, the welfare costs per ton in the $50–5% scenario were higher than that in the $50–1% scenario and typically by 20% to about 35%. In two cases, however, they are higher by 60% and 120%, both cases with among the highest costs. For the $25–5% and $25–1% trajectories with the same few models substantially outside this range, and again, mostly showing a slightly higher average cost per ton for the more rapidly rising carbon price.

### Welfare effects per ton of CO_2_ emissions — with capital tax recycling.

Across all tax and recycling scenarios, 36 of 39 scenarios found net present value costs per ton abated of $30 or less; 19 of 39 were $10 or less. The difference (generally) in average cost between household and capital tax recycling differs substantially among models, enough to change the rank order of models from lowest to highest cost. For example, the highest-cost model with household rebates became one of the lowest-cost models with capital tax recycling. Some showed very small differences, or in one case an increase. This suggests that, while there is general agreement that capital tax recycling reduces costs, the magnitude, mechanisms, and parameterization of these mechanisms are highly uncertain or at least differ substantially among the models.

### Welfare effects per ton of CO_2_ emissions — with labor tax recycling.

The average costs per ton of emissions reduced with labor tax recycling were generally intermediate to the results with household and capital tax recycling. In contrast to the capital tax recycling results, the rank order of models from lowest to highest cost is mostly consistent, except that the highest cost model dropped to second or third highest depending on the tax scenario. This suggests somewhat less difference in how this mechanism operates among the models.

As expected from the literature, welfare costs per ton are generally lowest with capital tax recycling.^15^ The exceptions are: EC-MSMR and NewERA, which show little difference between recycling options under all taxes; GH-E3, which shows little difference under the most stringent policy; and G-Cubed, for which lump-sum recycling provides better welfare impacts than capital tax recycling. In addition, for most models, there is little difference between rebates and labor tax recycling. The exceptions are DIEM and IGEM, both of which show lower welfare costs from labor tax recycling rather than lump-sum rebates. The differences are likely due to higher effective labor supply elasticities in those models.

## 4. How and Why Model Results Differ

The differences across models described in our results section stem from three broad sources: (1) how the electric sector is modeled; (2) the implicit supply elasticity of capital; and (3) the degree of foresight assumed in modeling savings.

In terms of the electric sector, the models fall into two groups. For NEMS, NewERA, and USREP-ReEDS, the carbon tax causes a relatively large shift between fuels in electricity generation and a relatively small change in the overall demand for electricity. Substitution between fuels, in other words, dominates the impact of the tax on overall electricity output. As a result, gas consumption rises relative to baseline during part or all of the 2020–2040 period. Fuel substitution will dominate the output effect when either or both of the following are true: the implicit elasticity of substitution between fuels is high, keeping the impact on electricity prices modest; or the elasticity of demand for electricity is low. NEMS and NewERA show relatively smaller emissions reductions than most models, which suggest that their electricity demand is relatively inelastic. USREP-ReEDS shows larger reductions, suggesting that it has relatively higher inter-fuel substitution. In all of the remaining models, the impact of the tax on electricity demand dominates the fuel-switching effect: the fall in electricity output is sufficient to lower overall gas demand even though utilities are switching out of coal and into gas. For those models, it appears that electricity demand is relatively elastic.

The second source of differences between the models is their apparent supply elasticity of capital. Most of the models show that capital tax recycling produces better welfare impacts than the alternatives after the first few years of the scenario. In those models, the reduction in capital taxation causes a significant expansion in the capital stock. Two exceptions are EC-MSMR and NewERA, both of which show little difference in welfare impacts across different recycling options. A likely cause is that those models have a relatively inelastic long-term supply of capital. The overall supply of capital to an economy is driven by domestic saving and international capital flows. For those two models, international capital flows may be constrained by assumptions imposed on the long-term balance of payments. Hence, it is likely that the two models have very inelastic long-term supplies of domestic savings.

Finally, the third difference between the models is the degree of foresight assumed on the part of agents. Models without foresight — EC-MSMR, FARM, NEMS, and USREP-ReEDS — show small impacts on GDP and its components, especially in the short run. Models with foresight typically show larger short run impacts on consumption, investment, or both. Among the models with foresight, ADAGE-US is unusual in that the carbon tax causes very little impact on investment in either the short or long run. At the opposite end of scale, the tax causes very large impacts on investment in G-Cubed, particularly in the short run.

Although we believe that these three broad sources account for many of the differences in the results, there are many other model differences that could also contribute to these differences (e.g., differences in cost assumptions across different technologies and sectors, assumptions about technological change, assumptions about capital adjustment costs) One might also think that differences in labor supply elasticities could be an important source of variation across models. However, most of the models have very similar welfare outcomes for the rebates and labor tax swaps. That suggests that most have very inelastic (or possibly fixed) labor supplies. The one model with the greatest differences between its rebate and labor tax swap scenarios is IGEM, which does indeed have a significant positive labor supply elasticity, and thus shows a larger benefit than other models from reducing the labor market distortion.

## 5. Conclusions

The EMF 32 study on carbon tax scenarios analyzed a set of illustrative policies in the United States that place an economy-wide tax on fossil-fuel-related carbon dioxide (CO_2_) emissions. Eleven modeling teams ran these stylized scenarios, which vary by the initial carbon tax rate, the rate at which the tax escalates over time, and the use of the revenues. This paper is a technical overview of the scenario design and the results for the effects of the four core policies. We compare various outcomes across the models, such as emissions, revenue, macroeconomic outcomes, sectoral impacts, and welfare.

Given the intent of this study, the most important conclusions of this study are for policy design. We refer the reader to Barron *et al.* (this issue) for a complete discussion of the implications of this study for policymakers. That paper enumerates the important robust takeaways as well as the caveats for this modeling project and other exercises like it. It also documents results not featured here, such as for scenarios in which modelers solve for the carbon tax paths that achieve particular emissions goals and the ways in which the carbon tax scenarios reduce conventional air pollutants. For more discussion of distributional outcomes of the policies, we refer the reader to Caron *et al.* (this issue). Macaluso *et al.* (this issue) review the sectoral and trade outcomes.

In short, the results here are consistent with much of the existing modeling literature on carbon pricing in the United States. Across all models, we find that the core carbon price scenarios lead to significant reductions in CO_2_ emissions, with the vast majority of the reductions occurring in the electricity sector and disproportionately through reductions in coal. Emissions reductions are largely independent of the uses of the revenues modeled here. Expected economic costs (not accounting for any of the benefits of GHG and conventional pollutant mitigation), in terms of either GDP or welfare, are modest, but they vary across models and policies. Using revenues to reduce preexisting capital or, to a lesser extent labor taxes, reduces welfare losses in most models relative to providing household rebates, but the magnitudes of the cost savings vary. The use of revenue can also have important distributional implications, as discussed in Macaluso *et al.* (this issue).

Future research can build on this work in several ways. First, as discussed in Barron *et al.* (this issue), the current version of the models involved in this study is not optimized for investigating deep decarbonization scenarios. In particular, they do not include the detailed treatment of the transportation sector to project deployment of technologies such as electric vehicles and low carbon biofuels that are essential to significant decarbonization of the sector. Secondly, the recent tax legislation in the United States substantially changes the statutory corporate tax rate and other revenue variables. This changes both baseline tax parameters and baseline debt projections, and this could change the efficiency gains from the tax swap scenarios. A useful extension would be to account for the new baseline and extend the scenarios to other options for revenue use, such as debt reduction and new spending, such as on infrastructure, research and development, or social safety net programs. Of course, one challenge for such work is how to investigate the potential macroeconomic and technology outcomes of new spending. But given the interest in these options, research in that direction would be valuable.

Thirdly, we emphasize the stylized and idealized nature of our policy scenarios in this study. One can easily imagine a more complex or less efficient evolution of policy. For example, disparate measures at the state and federal levels may overlap, and some sectors or regions may remain exempt. Research that elucidates when, where, and which policies are redundant and inefficient or usefully complementary would enrich the policy debate.

Finally, policymakers could benefit from research on the ways and outcomes of updating carbon tax trajectories, for example, in response to emissions or economic outcomes. For example, what are the tradeoffs of different measures that achieve particular emissions targets, either cumulative or annual, relative to approaches that provide more certainty for economic actors, such as those investing in new low-carbon technology? What does this mean for the optimal structure of climate commitments?

Certainly, the debate over how to price carbon best is not going away, but we believe that this study is an important contribution to the economic consensus that it can be done in a way that is both economically and environmentally responsible.

## Supplementary Material

Supplement 1

## Figures and Tables

**Table 1.** U.S. carbon tax policy scenarios.

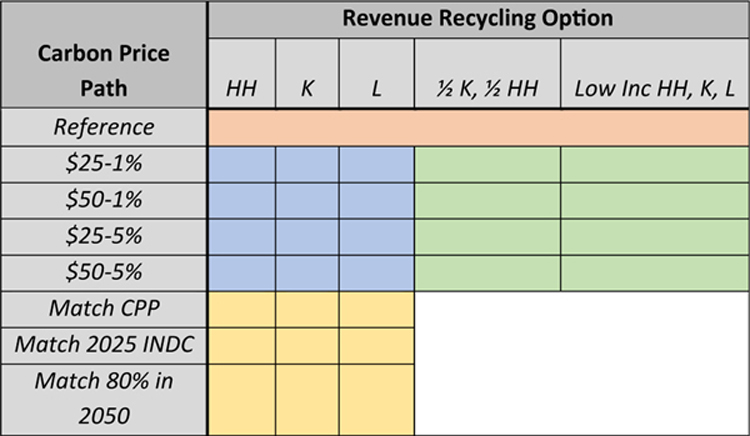

**Table 2.** Carbon price paths.

Carbon tax rate (in $2010 per metric ton CO_2_)	Description
Reference	No carbon tax
$25–1%	$25 in 2020, 1% real annual rate of increase
$50–1%	$50 in 2020, 1% real annual rate of increase
$25–5%	$25 in 2020, 5% real annual rate of increase
$50–5%	$50 in 2020, 5% real annual rate of increase

**Table 3.** Revenue recycling options.

Revenue recycling option	Description
*HH*	Revenue returned in lump-sum rebates to households
*K*	Revenue used to decrease capital income tax rates
L	Revenue used to decrease labor income tax rates
½ *K*, 1/*HH*	½ of revenue used to decrease capital income tax rates; ½ of revenue returned in lump-sum rebates to households
Low Inc. *HH*, *K*, *L*	Solve to provide lump-sum rebates to lowest quintile (by income) that leave welfare unchanged relative to baseline; ½ of remaining revenue used to decrease capital income tax rates; other ½ of remaining revenue used to decrease labor income tax rates

**Table 4.** Model attributes.

Model Name (institution/citation)	Regions	Covered sectors	Covered gases	Equilibrium approach	Intertemporal Solution approach	Model of tech. choice	Model base year	Model time step and horizon	Electric sector technology detail	Other important attributes
ADAGE-US (RTI/Woollacott)	U.S. only, 9 census regions	10 sectors, 5 nonenergy and 5 energy	CO_2_ only for EMF 32	CGE	Perfect foresight	CES	2010, based on 2013 data	5 year through 2050	Fuel-specific	Not running linked electricity model (EMA)
CEPE (ETH/Rausch et al.)	1 U.S.: small open economy of U.S.	5 energy (crude oil, refined oil, gas, coal, elec), 3 demand, 5 non-energy production	CO_2_ from fossil fuel combustion	CGE	Perfect foresight	CES	2015, based on 2011 data	5 year to 2165	3 fossil, hydro, nuclear, wind	Overlapping generations
DIEM (Duke/Ross)	CGE: 6 U.S., 8 global, Electricity: 48 U.S.	6 energy (crude oil, refined oil, ethanol, gas, coal, elec), 5 nonenergy, 3 demand, 6 nonenergy production	CO_2_, CH_4_, N_2_O, F-gas, SO_2_ , NOx, Hg	CGE: general, Electricity: partial	Perfect foresight	CGE: CES production function, Electricity: LP	2020	CGE: 5 years, 2060 Electricity: 2–5 years, 2060	CGE: 6 conventional (fossil, nuclear, hydro, biomass, wind, solar), 2 CCS (coal, gas)	Capital-energy structure for energy efficiency improvements;10 households per region
EC-MSMR (Env. CC Canada/Zhu et al.)	1 U.S., 15 non-U.S.	5 energy (crude oil, refined oil, gas, coal, electricity), 3 demand, 15 nonenergy production	CO_2_, CH_4_, N_2_ O, CGE F-gas		Recursive dynamic	CES production function	2011	5 years 2050	3 fossil (2 with and 3 without CCS), nuclear, hydro, wind, solar, biomass (w & w/o CCS), geothermal	8 crude oil and oil sands technologies for Canada, bitumen refinery technology for USA
FARM(USDA/Sands)	1 U.S.; 12 other world regions	38 production sectors (5 energy, 33 nonenergy)	CO_2_ from fossil fuel combust	CGE	Recursive dynamic	CES	2007	5-year to 2052	3 fossil (oil, gas, coal), nuclear, hydro, wind, solar PV, 2 bio-electricity (switchgrass, forest residue), MSW	CCS can be switched on or off for fossil and bio-electricity; land use by 18 agro-ecological zones and 9 crop types
G-Cubed (ANU, Syracuse, Brookings/ McKibben et al.)	1 U.S.; 8 other world regions	20 sectors; 14 energy, 6 nonenergy	CO_2_ from fossil fuel combust.	CGE	Perfect foresight	Nested CES	2015 but based on data through 2011	Annual to 2100	3 fossil (coal, gas, oil), nuclear, wind, solar, hydro, other	Mix of agents: some are liquidity constrained with myopic behavior
GH-E3 (RFF/Chen et al.)	1 U.S., 1 ROW	35 sectors, 9 energy, 26 nonenergy	CO_2_ from fossil fuel combust.	CGE	Perfect foresight	Nested CES	2013	1 year, 151 periods	3 generator types: coal, other fossil (primarily gas) and nonfossil whole-sale generators, with t/d sector to sell retail	Infinite-horizon model. Investment subject to adjustment costs.
IGEM-N (Northeastern, DJA/Jorgenson et al.)	1 U.S.: Open economy	NAICS-based: 6 energy (coal, oil mining, gas mining, refined petroleum, electric and gas utilities), 30 non-energy	CO_2_, CH_4_, N_2_O, F-gas, SO_2_ , NO_x_, Hg	CGE	Perfect foresight	Translog with Kalman filtering	Various. 2010 for model estimation	Annual 2015–2130	Fossil fuel energy inputs plus capital, labor and nonenergy materials	Demographic household welfare. Social welfare with inequality metrics
NEMS (EIA/Arora et al.)	1 U.S., 22 electricity market regions	4 supply (coal, oil, gas, renewables); electricity, refining; 4 demand-Res., Comm., Ind., Trans.	CO_2_ from fossil fuel combust. Elec sec SO_2_, NO_x_, Hg	Partial equilibrium with macro feedbacks	Myopic demand models; perfect foresight in electric sector	LP for electricity, with market sharing	Module-dependent, 2014 for electricity	Annual to 2040	Plant level detail for existing plants; New plants — PV, CSP, wind, coal, coal w/CCS, NG CT, NGCC, NGCC w/CCS, nuclear, hydro, GT, MSW, biomass; retrofits — coal to NG, coal w/CCS	CCS is 90% capture; endogenous capacity retirements
NewERA (NERA)	6 U.S. EMF Regions for macro model; 61 U.S. and 11 Canadian elec power pools	12 sectors: 6 energy (oil, gas, coal, refoil, ele, biofuels); 7 nonenergy sectors (ag., manuf., motor veh. manuf., energy int. sectors, services, trucking, oth comm. trans.)	CO_2_ for non-electric; CO_2_ , NO_x_, SO_2_ , and Hg for ele	Macro model — CGE; electric model — cost minimization (NLP)	Perfect fore-sight (full inter-tempo ral optimi zation)	Macro — CES; Electric mod el — technol ogy detailed LP	2015	3 years through 2049	7 fossil, 2 CCS (coal, gas), nuclear, 2 bio (landfill, bio-only), 6 renewable (hydro, geo, 2 wind, 3 solar), 2 storage (pump hydro, battery)	Linked model: top- down macro model fully linked with bottom-up electric sector model
USREP-ReEDS (NREL, MIT)	USREP: 6 U.S., 15 non-U.S. ReEDS: Elec sec 134 balancing areas	5 energy (crude oil, refined oil, gas, coal, elec) 3 demand, 6 nonenergy production	CO_2_-only for nonelec; elec sector includes CO_2_ , NO_x_, SO_2_ , Hg	CGE	Recursive dynamic (sequential solve)	CES in USREP for non-electricity sectors — LP in ReEDS elec sector	2006 (USREP), 2010 (ReEDS)	2 years through 2050	ReEDS: 7 fossil, 2 CCS (coal, gas), nuclear, 2 bio (landfill, bio-only), 5 renewable (hydro, geo, multiple wind and solar classes, technolo gies), 3 storage (pumped hydro, CAES, battery)	Linked model: top-down macro model fully linked with bottom-up electric sector model

**Table 5.** Estimated average haircut for core carbon tax scenarios, 2020–2040, with household rebates.

Scenario	ADAGE-US	CEPE	EC-MSMR	G-Cubed	NewERA
$25–1%	0.03	0.36	0.20	0.19	0.40
$50−1%	0.19	0.41	0.26	0.22	0.40
$25−5%	0.11	0.38	0.21	0.26	0.37
$50−5%	0.23	0.44	0.26		0.39

## Footnotes

1 See the introduction to this special issue (Fawcett *et al.*) for more information on how this study fits into the history of EMF studies related to climate policy, as well as for more information on how this special issue is organized. A second track of the EMF 32 study addresses technology and climate policy strategies for reducing emissions in the U.S. electric power sector (Murray *et al*., 2018)

2 Leiserowitz *et al.* (2017).

3 Quinnipiac (2017).

4 Holtz-Eakin *et al.* (2017) and Carbon Leadership Council (2017).

5 Limiting the policy to fossil energy CO_2_ is a simplifying assumption used for this study. See the EMF 21 study on multi-greenhouse gas mitigation and climate policy (Weyant *et al.*, 2006) for an extensive discussion on the impact of including non-CO_2_ gases and land use emissions in climate policy. The EMF 24 study on U.S. technology and climate policy strategies (Fawcett *et al.*, 2014) also examines a range of U.S. climate policies that include non-CO_2_ and land use emissions.

6 One modeling group explored the effects of applying a border carbon adjustment on imported goods. See McKibbin *et al.* (this issue) For an extensive examination of border carbon adjustments, see the EMF 29 study on the role of border carbon adjustments in unilateral climate policy (Böhringer *et al.*, 2012).

7 Whitehouse (2017).

8 Larson (2017).

9 Some models only report fossil fuel production. Where wind, solar, hydro and nuclear are available, the values shown are direct equivalents.

10 Under budgeting rules that go back to the Congressional Budget Act of 1974 (PL 93–344), the JCT estimates the revenue effects of all tax legislation. The JCT scores the net revenue effects of a tax change after taking account of its impact on revenues from other taxes. For excise taxes like a carbon tax, JCT assumes revenues from income and payroll taxes fall by a set percentage (called the offset) of the revenue from the excise tax. JCT does not consider impacts on state tax revenue, which some analysts calculate endogenously in their models. JCT (1970) describes its role in the budget process, JCT (2017) describes its revenue estimating process, and JCT (2016) gives the rationale and procedure for the offset.

11 JCT (2016).

12 As shown in Table 4, the perfect foresight models are ADAGE-US, CEPE, DIEM, G-Cubed, GH-E3, IGEM, and NewERA.

13 Mas-Colell *et al.* (1995).

14 Unscaled cumulative EV’s appear in the supplementary material.

15 See five illustrative papers in the March 2015 issue of the *National Tax Journal*: Jorgenson *et al.* (2015); McKibbin *et al.* (2015); Rausch and Reilly (2015); Tuladhar *et al.* (2015); and Williams *et al.* (2015).
