# Supplementary material for: OVERVIEW OF THE EMF 32 STUDY ON U.S. CARBON TAX SCENARIOS
Source: Clim Chang Econ (Singap). Author manuscript; Available in PMC 2019 Dec 16. (PMC6913042; doi:10.1142/S201000781840002X)
Supplement: Supplement 1 [file NIHMS1021974-supplement-Supplement_1.docx]

**Supplementary Materials**

1. **Supporting Graphics and Analysis**


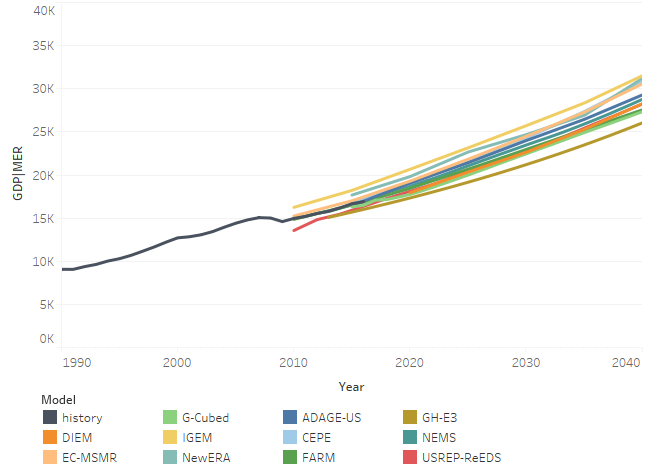


**Figure S1. Reference GDP projections (billion 2010$)**


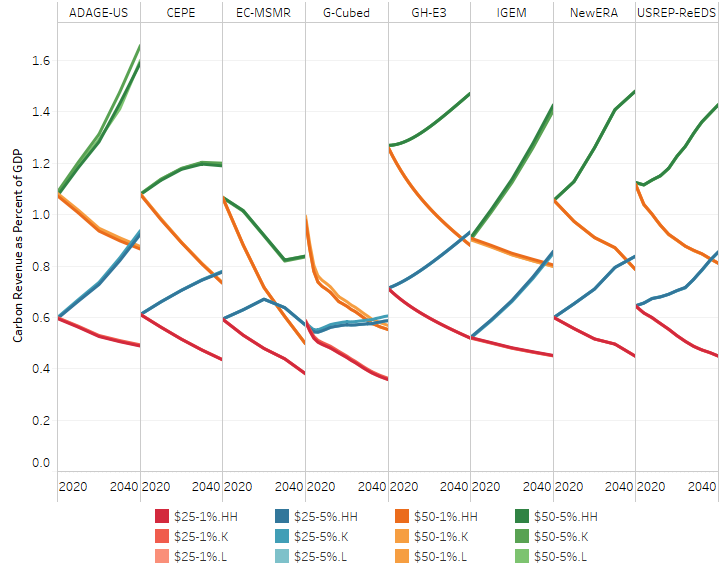


**Figure S2. Carbon revenue as a percentage of GDP**


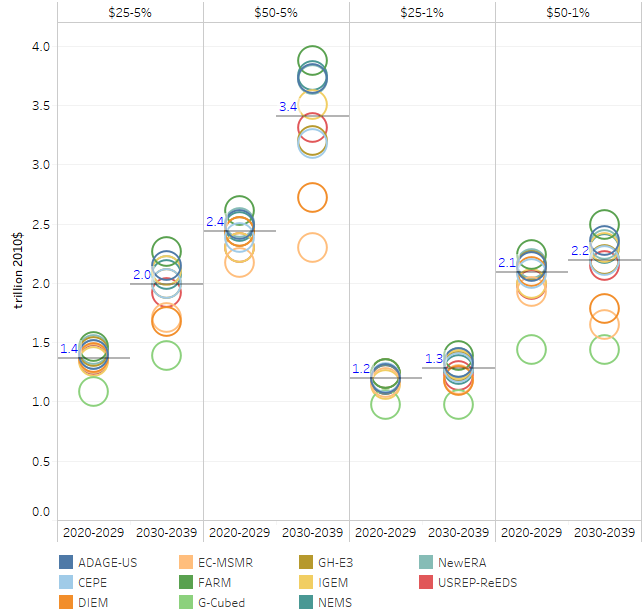


Figure S3. Cumulative gross carbon tax revenue across two 10-year periods

Revenue estimators generally aggregate the effects of any new revenue policy into 10-year budget windows. Figure S3 shows the total gross carbon tax revenue in the periods 2020-2029 and 2030-2039 in trillions of real 2010 dollars. These estimates differ from the approach used by official scoring agencies such as the Joint Committee on Taxation in that they do not account for any reductions in revenues from other instruments and they are in constant real terms.


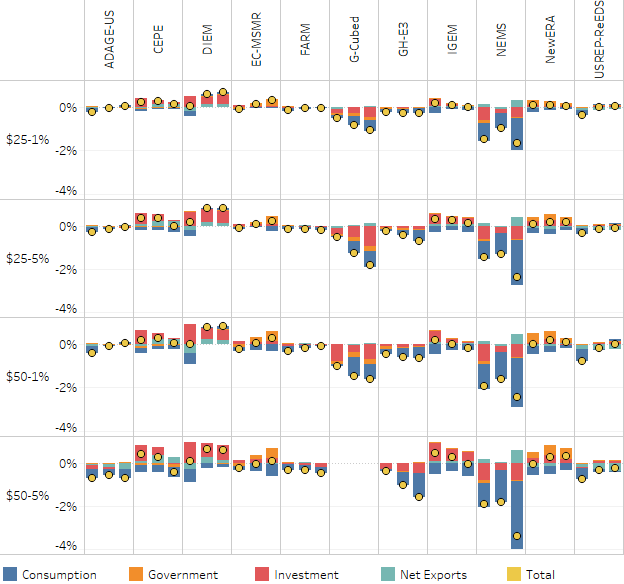


Figure S4a. Percent change in GDP and its components relative to reference total GDP in 2020, 2030, and 2040, capital income tax recycling


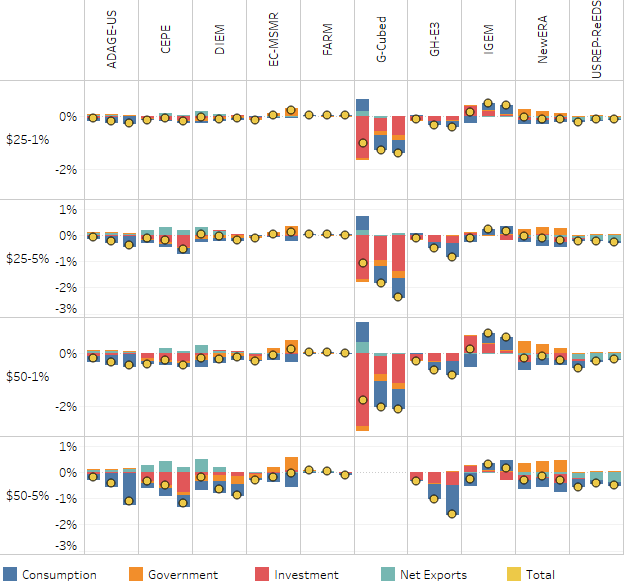


Figure S4b. Percent change in GDP and its components relative to reference total GDP in 2020, 2030, and 2040, labor income tax recycling.


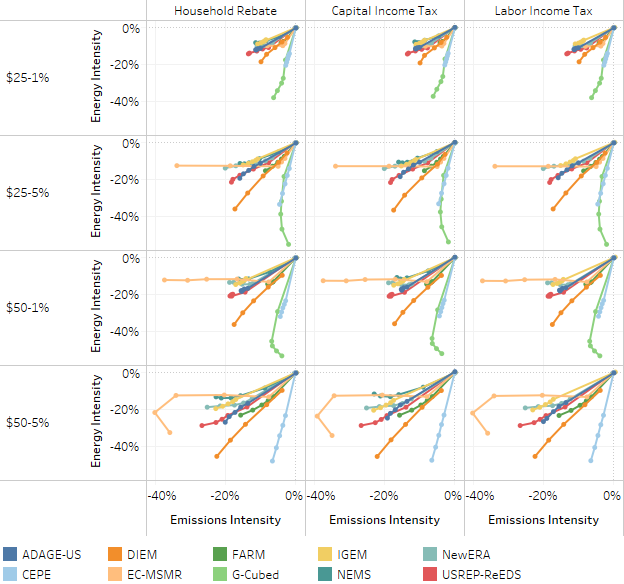


**Figure S5. Primary energy intensity and emissions intensity percent changes, 2015-2040.**


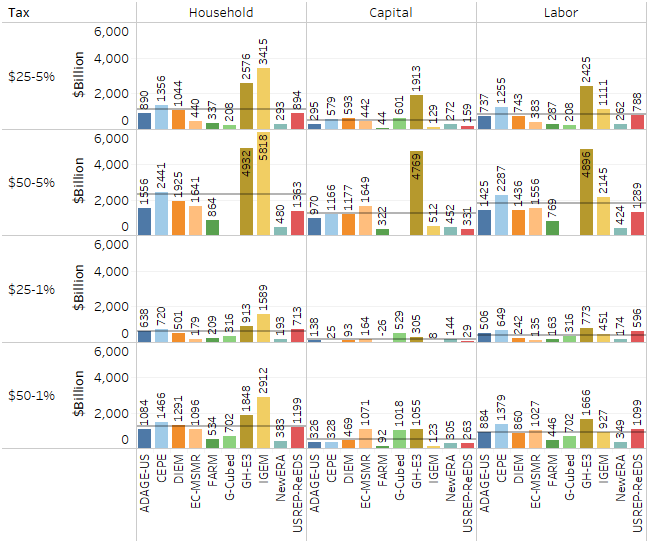


**Figure S6. Cumulative aggregate welfare reduction (EV) across core scenarios from 2020 to 2040. Mean represented by straight line.**

Figure S6 presents cumulative aggregate losses in welfare, as measured by equivalent variations (EVs), for each of the core carbon tax policies. All model results show net negative effects on net present value of welfare (measured as equivalent variation) across call carbon tax scenarios and all revenue recycling approaches, with the exception of one model’s results with capital tax recycling, which showed recycling to just balance or slightly offset abatement costs. The welfare impacts over time varied significantly among models.


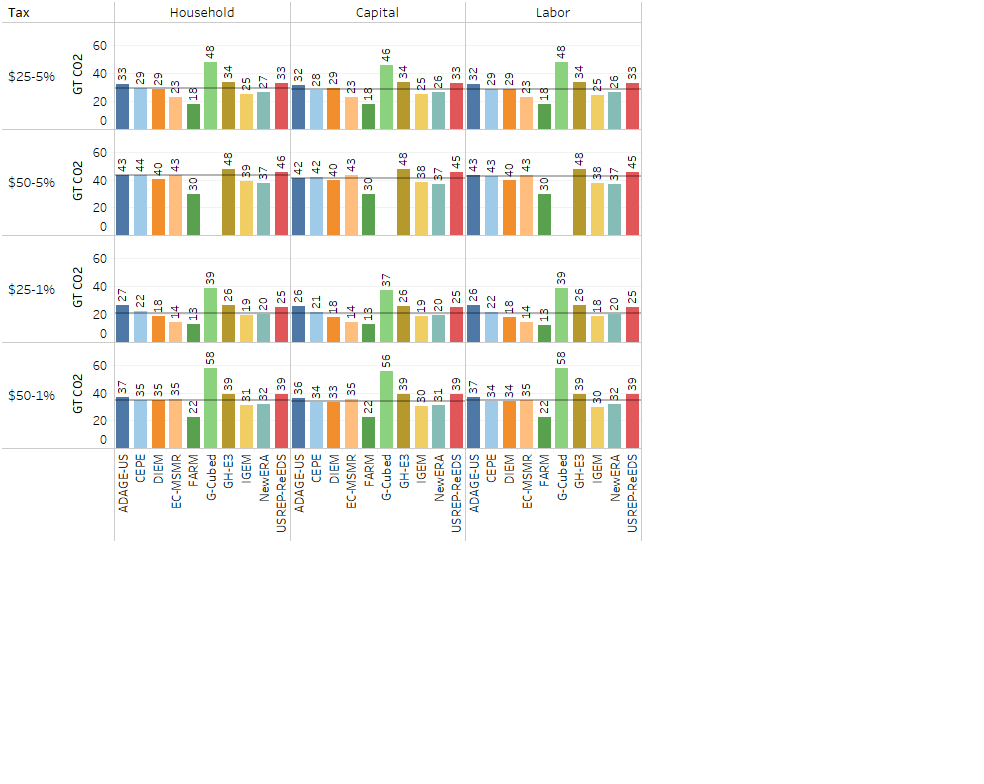


**Figure S7. Cumulative emission reductions across tax and revenue recycling scenarios. Mean represented by straight line.**


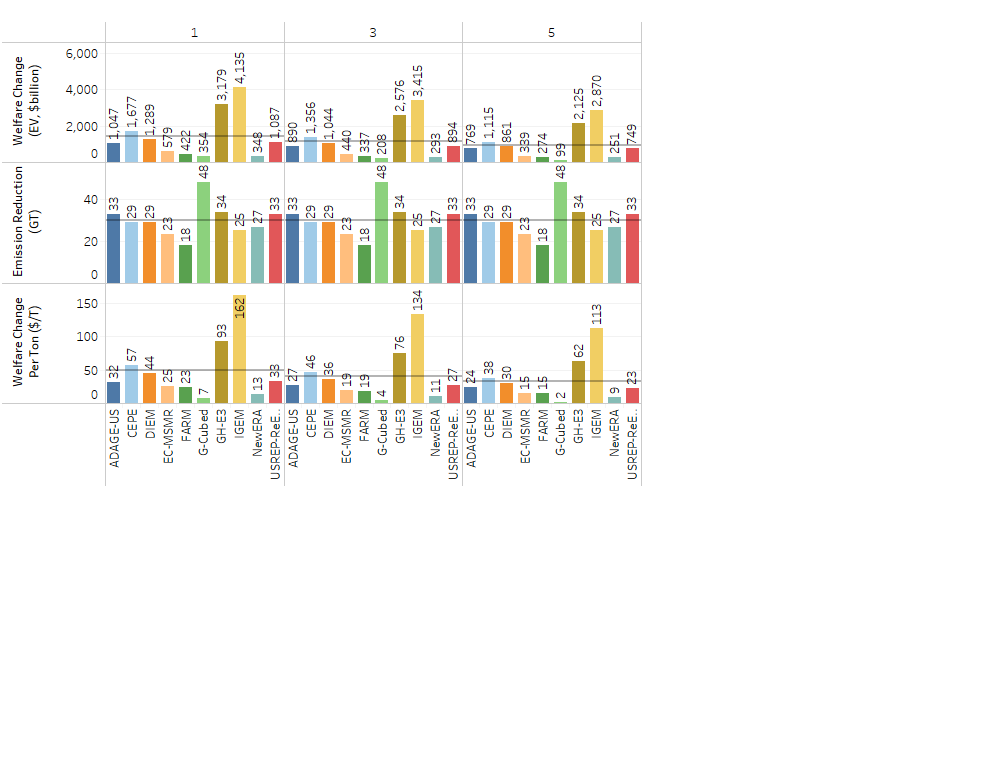


**Figure S8. Welfare reduction, emission reductions, and welfare reduction per ton abated at discount rates of 1, 3, and 5% for the $25-5% scenario rebates to households, 2020-2040. Mean represented by straight line.**

1. **Description of Additional Scenarios**
   1. *Distributional Outcome Scenarios*

The half capital tax swap/half rebate scenarios reflect one possible way to balance efficiency-enhancing revenue recycling with a progressive distribution of the carbon tax revenue. Two other scenarios help elucidate ways to ensure low-income households are held harmless but also provide at least a weak double dividend. In one of these runs, modelers solve to provide transfers to lowest quintile (by income) to leave welfare unchanged relative to baseline; evenly split the remaining revenue between decreases in the L Tax Rate and the K Tax Rate. In the other, they determine any combination(s) of rebates and tax swaps that maximize welfare subject to the conditions that the program (1) holds welfare of the lowest quintile (by income and/or consumption) constant relative to baseline (or higher) and (2) is progressive through all quintiles.

*2.2 Environmental Target Scenarios*

In *Match CPP* environmental target scenario, modelers solve for a carbon tax imposed only on fossil fuels used in the electric power sector such that emissions match EPA’s expected outcome of the Clean Power Plan in 2030. The tax starts in 2020 and rises at 5 percent over inflation until 2030, then remains constant in real terms thereafter. As documented in the final rule’s Regulatory Impact Analysis (RIA), the EPA estimates that the Mass-Based Approach will result in emissions of 1709 million short tons (1551 million metric tons) of CO_2_ in the electricity sector in 2030, a decline of 518 million short tons relative to the base case projection of 2,227 million short tons. ^^[[1]](#footnote-2)^^

In the *Match 2025 INDC* scenario, modelers solve for the carbon tax trajectories consistent with the intended nationally determined contribution (INDC) that the U.S. communicated to the United Nations Framework Convention on Climate Change (UNFCCC) in 2015.^[[2]](#footnote-3),^^[[3]](#footnote-4)^ The 2015 U.S. INDC submission included an economy-wide emissions reduction target of 26 to 28 percent below its net 2005 emissions level in 2025.

In the *Match 80% in 2050* scenario, modelers solve for the carbon tax trajectories consistent with both the 2025 target from the *Match 2025 INDC* scenario, and a notional 80% below 2005 net emissions target for 2050. The goals for all of the environmental target scenarios do not cap cumulative emissions, just match the specified annual emissions level in the particular out year with a carbon tax that begins in 2020 and rises at a consistent rate.

Given our focus here on fossil energy CO_2_, and not all of the participating models include non-CO2 GHGs or land use emissions, we convert the goals in the *Match 2025 INDC* scenario and the *Match 80% in 2050* scenario for net GHG emissions (including terrestrial carbon and non-CO_2_ GHGs) into goals for gross fossil energy CO_2_ emissions. Table 2 below shows the full array of U.S. GHG sources and sinks in the 2005 base year, along with their counterparts for 2012 for comparison.^[[4]](#footnote-5)^

**Table 2. U.S. GHG inventory (in MtCO_2_e)**

| Gas/Source | 2005 | 2012 |
| --- | --- | --- |
| Fossil Fuel Combustion | 5,752.9 | 5,072.3 |
| Other CO_2_ | 359.3 | 310.9 |
| Non-CO_2_ | 1,141.6 | 1,142.4 |
| Land Use, Land-Use Change, and Forestry (Sink) | (1,030.7) | (979.3) |
| Total | 7,253.8 | 6,525.6 |
| Net Emissions (Sources and Sinks) | 6,223.1 | 5,546.3 |

To translate goals for reductions of net GHG emissions into goals for reductions in gross fossil CO_2_ emissions, we first calculate the reduction in net emissions (in MtCO_2_e) implied by the percentage reduction goals of 26% and 80%. Assuming an exogenous land-related sink of -1,000 MtCO_2_e in 2025 and -500 MtCO_2_e in 2050,^[[5]](#footnote-6)^ we determine that target U.S. net emissions for 2025 and 2050 are 4,605 and 1,245 respectively, as shown in the last row of Table 3 below.

**Table 3. Fossil Carbon Abatement Consistent with Net GHG Goals (in MtCO_2_e)**

|  | ***Inventory*** | ***Projections / Targets*** | | | ***% below 2005*** | | |
| --- | --- | --- | --- | --- | --- | --- | --- |
| ***Gas/Source*** | **2005** | | **2025** | **2050** | | **2025** | **2050** |
| *Fossil Fuel Combustion* | 5,753 | | 4,445 | 1,384 | | -23% | -76% |
| *Other CO2* | 359 | | 278 | 86 | | -23% | -76% |
| *Non-CO2* | 1,142 | | 882 | 275 | | -23% | -76% |
| *Land Use, Land-Use Change, and Forestry (Sink)* | -1,031 | | -1,000 | -500 | | -3% | -51% |
| *Total* | 7,254 | | 5,605 | 1,745 | | -23% | -76% |
| *Net Emissions (Sources and Sinks)* | 6,223 | | **4,605** | **1,245** | | **-26%** | **80%** |

For convenience, we assume a target for gross fossil CO_2_ emissions that reflects the same percentage point decline as the net emissions reductions target for the entire U.S. GHG inventory. As shown in Table 3, that means that each gross source category reduces emissions by 23% in 2025 and 76% in 2050. For fossil carbon, shown highlighted in yellow, target emissions are 4,445 and 1,384 in 2025 and 2050, respectively.

Because they do not cover all sources and gases, these scenarios do not represent the most efficient policy design, nor do they exactly represent what it would take to meet the U.S. 2025 and 2050 emissions goals. Rather, they give illustrative insights into the ambition associated with the fossil energy component of those goals in a way that is consistent with the other carbon tax scenarios in the study.

In the *Match 2025 INDC* scenario, modelers focus an emissions target for 2025. They solve for the initial (2020) value (in 2010$/tCO_2_) of a carbon tax that applies to fossil energy CO_2_ only, starts in 2020, rises at 5% real per year until 2050, then remains constant in real terms. The trajectory achieves an annual level of fossil energy CO_2_ emissions in 2025 of 4,445 mtCO_2_.

In the *Match 80% in 2050* scenario, modelers solve for initial carbon tax rate and real growth rate so as to hit 26% reduction target relative to 2005 in 2025 and 80 percent reduction target relative to 2005 in 2050. That means fossil energy CO_2_ emissions in 2025 of 4,445 mtCO_2_ and 1,384 mtCO_2_ in 2050.

The EMF 32 exercise also included a scenario that imposed carbon prices equal to the social cost of carbon in each year starting in 2020. Since the carbon price in this scenario is very similar to the carbon price in the *$50-1%* scenario, this scenario is not included in any of the overview papers.

1. <https://www.epa.gov/cleanpowerplantoolbox/clean-power-plan-documents-and-resources>; <https://www.epa.gov/sites/production/files/2015-08/copy_of_cpp_final_goals_and_nscomplement_summary_table_8-4_0.xlsx> [↑](#footnote-ref-2)
2. The United States submission to the Paris agreement appears here: <http://www4.unfccc.int/submissions/INDC/Published%20Documents/United%20States%20of%20America/1/U.S.%20Cover%20Note%20INDC%20and%20Accompanying%20Information.pdf>. [↑](#footnote-ref-3)
3. The President of the United States announced on June 1, 2017 that “the United States will withdraw from the Paris climate accord…But begin negotiations to re-enter either the Paris accord or really entirely new transaction....” The EMF 32 scenarios were designed before this decision to withdraw from the Paris Agreement. While the 2015 U.S. INDC submission is no longer official U.S. policy, for the purpose of this exercise, the *Match 2025 INDC* scenario still provides a useful way to compare the carbon price trajectories required by different models to meet a specific quantity target. [↑](#footnote-ref-4)
4. EPA. (2014). Inventory of U.S. greenhouse gas emissions and sinks: 1990-2014. Retrieved from < https://www.epa.gov/ghgemissions/inventory-us-greenhouse-gas-emissions-and-sinks-1990-2012> [↑](#footnote-ref-5)
5. For comparison, the 2016 U.S. Biennial Report included a high and low projection for *the Land Use, Land-Use Change and Forestry (Sink)* that spanned the range from -908 to -1,201 in 2025 and -689 to -1,118 in 2030. [↑](#footnote-ref-6)
